# Supplementary material for: Overlapping cell population expression profiling and regulatory inference in C. elegans
Source: BMC Genomics. 2016 Feb 29;17:159. doi: 10.1186/s12864-016-2482-z (PMC4772325; doi:10.1186/s12864-016-2482-z)
Supplement: Additional file 13: — Web supplement. (DOC 21 kb) [file 12864_2016_2482_MOESM13_ESM.zip › sortWeb/clusters/hier.300.clusters/96.html]

Cluster 96 

## Cluster 96

### Expression

| cnd-1 rep. 1 | cnd-1 rep. 2 | cnd-1 rep. 3 | pha-4 rep. 1 | pha-4 rep. 2 | pha-4 rep. 3 | ceh-27 | ceh-36 | ceh-6 | F21D5.9 | mir-57 | mls-2 | pal-1 | pros-1 | ttx-3 | unc-130 | hlh-16 | irx-1 | ceh-6 (+) hlh-16 (+) | ceh-6 (+) hlh-16 (-) | ceh-6 (-) hlh-16 (+) | cnd-1 singlets | pha-4 singlets | 0 | 60 | 120 | 150 | 180 | 240 | 330 | 390 | 420 | 480 | 540 | 570 | 600 | 630 | 660 | NAME | Functional description |
| --- | --- | --- | --- | --- | --- | --- | --- | --- | --- | --- | --- | --- | --- | --- | --- | --- | --- | --- | --- | --- | --- | --- | --- | --- | --- | --- | --- | --- | --- | --- | --- | --- | --- | --- | --- | --- | --- | --- | --- |
|  |  |  |  |  |  |  |  |  |  |  |  |  |  |  |  |  |  |  |  |  |  |  |  |  |  |  |  |  |  |  |  |  |  |  |  |  |  | C13A2.3 |  |
|  |  |  |  |  |  |  |  |  |  |  |  |  |  |  |  |  |  |  |  |  |  |  |  |  |  |  |  |  |  |  |  |  |  |  |  |  |  | *str-57* | Seven TM Receptor |
|  |  |  |  |  |  |  |  |  |  |  |  |  |  |  |  |  |  |  |  |  |  |  |  |  |  |  |  |  |  |  |  |  |  |  |  |  |  | *tag-140* | Temporarily Assigned Gene name |
|  |  |  |  |  |  |  |  |  |  |  |  |  |  |  |  |  |  |  |  |  |  |  |  |  |  |  |  |  |  |  |  |  |  |  |  |  |  | *lgc-55* | Ligand-Gated ion Channel |
|  |  |  |  |  |  |  |  |  |  |  |  |  |  |  |  |  |  |  |  |  |  |  |  |  |  |  |  |  |  |  |  |  |  |  |  |  |  | *arrd-15* | ARRestin Domain protein |
|  |  |  |  |  |  |  |  |  |  |  |  |  |  |  |  |  |  |  |  |  |  |  |  |  |  |  |  |  |  |  |  |  |  |  |  |  |  | *glb-11* | GLoBin related |
|  |  |  |  |  |  |  |  |  |  |  |  |  |  |  |  |  |  |  |  |  |  |  |  |  |  |  |  |  |  |  |  |  |  |  |  |  |  | C18G1.1 |  |
|  |  |  |  |  |  |  |  |  |  |  |  |  |  |  |  |  |  |  |  |  |  |  |  |  |  |  |  |  |  |  |  |  |  |  |  |  |  | T13C5.9 |  |
|  |  |  |  |  |  |  |  |  |  |  |  |  |  |  |  |  |  |  |  |  |  |  |  |  |  |  |  |  |  |  |  |  |  |  |  |  |  | *ceh-33* | C. Elegans Homeobox |
|  |  |  |  |  |  |  |  |  |  |  |  |  |  |  |  |  |  |  |  |  |  |  |  |  |  |  |  |  |  |  |  |  |  |  |  |  |  | T03G11.9 |  |
|  |  |  |  |  |  |  |  |  |  |  |  |  |  |  |  |  |  |  |  |  |  |  |  |  |  |  |  |  |  |  |  |  |  |  |  |  |  | C44H4.6 |  |
|  |  |  |  |  |  |  |  |  |  |  |  |  |  |  |  |  |  |  |  |  |  |  |  |  |  |  |  |  |  |  |  |  |  |  |  |  |  | *zig-6* | 2 (Zwei) IG domain protein |
|  |  |  |  |  |  |  |  |  |  |  |  |  |  |  |  |  |  |  |  |  |  |  |  |  |  |  |  |  |  |  |  |  |  |  |  |  |  | *lron-12* | eLRR (extracellular Leucine-Rich Repeat) ONly |
|  |  |  |  |  |  |  |  |  |  |  |  |  |  |  |  |  |  |  |  |  |  |  |  |  |  |  |  |  |  |  |  |  |  |  |  |  |  | *clec-264* | C-type LECtin |
|  |  |  |  |  |  |  |  |  |  |  |  |  |  |  |  |  |  |  |  |  |  |  |  |  |  |  |  |  |  |  |  |  |  |  |  |  |  | *cth-1* | CystaTHionine gamma lyase |
|  |  |  |  |  |  |  |  |  |  |  |  |  |  |  |  |  |  |  |  |  |  |  |  |  |  |  |  |  |  |  |  |  |  |  |  |  |  | *lev-8* | LEVamisole resistant |
|  |  |  |  |  |  |  |  |  |  |  |  |  |  |  |  |  |  |  |  |  |  |  |  |  |  |  |  |  |  |  |  |  |  |  |  |  |  | ZK809.8 |  |
|  |  |  |  |  |  |  |  |  |  |  |  |  |  |  |  |  |  |  |  |  |  |  |  |  |  |  |  |  |  |  |  |  |  |  |  |  |  | F56C3.9 |  |
|  |  |  |  |  |  |  |  |  |  |  |  |  |  |  |  |  |  |  |  |  |  |  |  |  |  |  |  |  |  |  |  |  |  |  |  |  |  | T12B3.7 |  |
|  |  |  |  |  |  |  |  |  |  |  |  |  |  |  |  |  |  |  |  |  |  |  |  |  |  |  |  |  |  |  |  |  |  |  |  |  |  | *col-185* | COLlagen |
|  |  |  |  |  |  |  |  |  |  |  |  |  |  |  |  |  |  |  |  |  |  |  |  |  |  |  |  |  |  |  |  |  |  |  |  |  |  | *pxl-1* | PaXiLlin homolog |
|  |  |  |  |  |  |  |  |  |  |  |  |  |  |  |  |  |  |  |  |  |  |  |  |  |  |  |  |  |  |  |  |  |  |  |  |  |  | D1005.t1 |  |
|  |  |  |  |  |  |  |  |  |  |  |  |  |  |  |  |  |  |  |  |  |  |  |  |  |  |  |  |  |  |  |  |  |  |  |  |  |  | F23D12.7 |  |
|  |  |  |  |  |  |  |  |  |  |  |  |  |  |  |  |  |  |  |  |  |  |  |  |  |  |  |  |  |  |  |  |  |  |  |  |  |  | *pvf-1* | PDGF/VEGF growth factor related |
|  |  |  |  |  |  |  |  |  |  |  |  |  |  |  |  |  |  |  |  |  |  |  |  |  |  |  |  |  |  |  |  |  |  |  |  |  |  | W08A12.2 |  |
|  |  |  |  |  |  |  |  |  |  |  |  |  |  |  |  |  |  |  |  |  |  |  |  |  |  |  |  |  |  |  |  |  |  |  |  |  |  | *idhg-2* | Isocitrate DeHydrogenase Gamma |
|  |  |  |  |  |  |  |  |  |  |  |  |  |  |  |  |  |  |  |  |  |  |  |  |  |  |  |  |  |  |  |  |  |  |  |  |  |  | Y53H1B.2 |  |
|  |  |  |  |  |  |  |  |  |  |  |  |  |  |  |  |  |  |  |  |  |  |  |  |  |  |  |  |  |  |  |  |  |  |  |  |  |  | *ttr-5* | TransThyretin-Related family domain |
|  |  |  |  |  |  |  |  |  |  |  |  |  |  |  |  |  |  |  |  |  |  |  |  |  |  |  |  |  |  |  |  |  |  |  |  |  |  | *ceeh-2* | Caenorhabditis EpoxidE Hydrolase |
|  |  |  |  |  |  |  |  |  |  |  |  |  |  |  |  |  |  |  |  |  |  |  |  |  |  |  |  |  |  |  |  |  |  |  |  |  |  | *maa-1* | Membrane Associated Acyl-CoA binding protein |
|  |  |  |  |  |  |  |  |  |  |  |  |  |  |  |  |  |  |  |  |  |  |  |  |  |  |  |  |  |  |  |  |  |  |  |  |  |  | *ttr-48* | TransThyretin-Related family domain |
|  |  |  |  |  |  |  |  |  |  |  |  |  |  |  |  |  |  |  |  |  |  |  |  |  |  |  |  |  |  |  |  |  |  |  |  |  |  | *glna-2* | GLutamiNAse |
|  |  |  |  |  |  |  |  |  |  |  |  |  |  |  |  |  |  |  |  |  |  |  |  |  |  |  |  |  |  |  |  |  |  |  |  |  |  | *kel-8* | KELch-repeat containing protein |
|  |  |  |  |  |  |  |  |  |  |  |  |  |  |  |  |  |  |  |  |  |  |  |  |  |  |  |  |  |  |  |  |  |  |  |  |  |  | C08B6.4 |  |
|  |  |  |  |  |  |  |  |  |  |  |  |  |  |  |  |  |  |  |  |  |  |  |  |  |  |  |  |  |  |  |  |  |  |  |  |  |  | *sqv-4* | SQuashed Vulva |
|  |  |  |  |  |  |  |  |  |  |  |  |  |  |  |  |  |  |  |  |  |  |  |  |  |  |  |  |  |  |  |  |  |  |  |  |  |  | *best-8* | BESTrophin (chloride channel) homolog |
|  |  |  |  |  |  |  |  |  |  |  |  |  |  |  |  |  |  |  |  |  |  |  |  |  |  |  |  |  |  |  |  |  |  |  |  |  |  | F13H8.5 |  |
|  |  |  |  |  |  |  |  |  |  |  |  |  |  |  |  |  |  |  |  |  |  |  |  |  |  |  |  |  |  |  |  |  |  |  |  |  |  | R07E3.6 |  |
|  |  |  |  |  |  |  |  |  |  |  |  |  |  |  |  |  |  |  |  |  |  |  |  |  |  |  |  |  |  |  |  |  |  |  |  |  |  | R07E5.4 |  |
|  |  |  |  |  |  |  |  |  |  |  |  |  |  |  |  |  |  |  |  |  |  |  |  |  |  |  |  |  |  |  |  |  |  |  |  |  |  | T03F6.4 |  |
|  |  |  |  |  |  |  |  |  |  |  |  |  |  |  |  |  |  |  |  |  |  |  |  |  |  |  |  |  |  |  |  |  |  |  |  |  |  | F31D4.5 |  |
|  |  |  |  |  |  |  |  |  |  |  |  |  |  |  |  |  |  |  |  |  |  |  |  |  |  |  |  |  |  |  |  |  |  |  |  |  |  | *ptr-16* | PaTched Related family |
|  |  |  |  |  |  |  |  |  |  |  |  |  |  |  |  |  |  |  |  |  |  |  |  |  |  |  |  |  |  |  |  |  |  |  |  |  |  | T06A4.3 |  |
|  |  |  |  |  |  |  |  |  |  |  |  |  |  |  |  |  |  |  |  |  |  |  |  |  |  |  |  |  |  |  |  |  |  |  |  |  |  | H10E21.4 |  |
|  |  |  |  |  |  |  |  |  |  |  |  |  |  |  |  |  |  |  |  |  |  |  |  |  |  |  |  |  |  |  |  |  |  |  |  |  |  | *cutl-14* | CUTiclin-Like |
|  |  |  |  |  |  |  |  |  |  |  |  |  |  |  |  |  |  |  |  |  |  |  |  |  |  |  |  |  |  |  |  |  |  |  |  |  |  | Y34B4A.10 |  |
|  |  |  |  |  |  |  |  |  |  |  |  |  |  |  |  |  |  |  |  |  |  |  |  |  |  |  |  |  |  |  |  |  |  |  |  |  |  | *col-144* | COLlagen |
|  |  |  |  |  |  |  |  |  |  |  |  |  |  |  |  |  |  |  |  |  |  |  |  |  |  |  |  |  |  |  |  |  |  |  |  |  |  | *col-182* | COLlagen |
|  |  |  |  |  |  |  |  |  |  |  |  |  |  |  |  |  |  |  |  |  |  |  |  |  |  |  |  |  |  |  |  |  |  |  |  |  |  | C27H5.4 |  |
|  |  |  |  |  |  |  |  |  |  |  |  |  |  |  |  |  |  |  |  |  |  |  |  |  |  |  |  |  |  |  |  |  |  |  |  |  |  | *cnc-8* | CaeNaCin (Caenorhabditis bacteriocin) |
|  |  |  |  |  |  |  |  |  |  |  |  |  |  |  |  |  |  |  |  |  |  |  |  |  |  |  |  |  |  |  |  |  |  |  |  |  |  | *col-104* | COLlagen |
|  |  |  |  |  |  |  |  |  |  |  |  |  |  |  |  |  |  |  |  |  |  |  |  |  |  |  |  |  |  |  |  |  |  |  |  |  |  | *col-77* | COLlagen |
|  |  |  |  |  |  |  |  |  |  |  |  |  |  |  |  |  |  |  |  |  |  |  |  |  |  |  |  |  |  |  |  |  |  |  |  |  |  | *col-167* | COLlagen |
|  |  |  |  |  |  |  |  |  |  |  |  |  |  |  |  |  |  |  |  |  |  |  |  |  |  |  |  |  |  |  |  |  |  |  |  |  |  | *ram-2* | abnormal RAy Morphology |
|  |  |  |  |  |  |  |  |  |  |  |  |  |  |  |  |  |  |  |  |  |  |  |  |  |  |  |  |  |  |  |  |  |  |  |  |  |  | *col-107* | COLlagen |
|  |  |  |  |  |  |  |  |  |  |  |  |  |  |  |  |  |  |  |  |  |  |  |  |  |  |  |  |  |  |  |  |  |  |  |  |  |  | *col-3* | COLlagen |
|  |  |  |  |  |  |  |  |  |  |  |  |  |  |  |  |  |  |  |  |  |  |  |  |  |  |  |  |  |  |  |  |  |  |  |  |  |  | C29G2.6 |  |
|  |  |  |  |  |  |  |  |  |  |  |  |  |  |  |  |  |  |  |  |  |  |  |  |  |  |  |  |  |  |  |  |  |  |  |  |  |  | R12E2.14 |  |
|  |  |  |  |  |  |  |  |  |  |  |  |  |  |  |  |  |  |  |  |  |  |  |  |  |  |  |  |  |  |  |  |  |  |  |  |  |  | C02E7.7 |  |
|  |  |  |  |  |  |  |  |  |  |  |  |  |  |  |  |  |  |  |  |  |  |  |  |  |  |  |  |  |  |  |  |  |  |  |  |  |  | R12E2.15 |  |
|  |  |  |  |  |  |  |  |  |  |  |  |  |  |  |  |  |  |  |  |  |  |  |  |  |  |  |  |  |  |  |  |  |  |  |  |  |  | C02E7.6 |  |
|  |  |  |  |  |  |  |  |  |  |  |  |  |  |  |  |  |  |  |  |  |  |  |  |  |  |  |  |  |  |  |  |  |  |  |  |  |  | *cpg-7* | Chondroitin ProteoGlycan |
|  |  |  |  |  |  |  |  |  |  |  |  |  |  |  |  |  |  |  |  |  |  |  |  |  |  |  |  |  |  |  |  |  |  |  |  |  |  | *cut-2* | CUTiclin |
|  |  |  |  |  |  |  |  |  |  |  |  |  |  |  |  |  |  |  |  |  |  |  |  |  |  |  |  |  |  |  |  |  |  |  |  |  |  | *col-65* | COLlagen |
|  |  |  |  |  |  |  |  |  |  |  |  |  |  |  |  |  |  |  |  |  |  |  |  |  |  |  |  |  |  |  |  |  |  |  |  |  |  | Y47D7A.13 |  |
|  |  |  |  |  |  |  |  |  |  |  |  |  |  |  |  |  |  |  |  |  |  |  |  |  |  |  |  |  |  |  |  |  |  |  |  |  |  | F23H12.7 |  |
|  |  |  |  |  |  |  |  |  |  |  |  |  |  |  |  |  |  |  |  |  |  |  |  |  |  |  |  |  |  |  |  |  |  |  |  |  |  | T21G5.6 |  |
|  |  |  |  |  |  |  |  |  |  |  |  |  |  |  |  |  |  |  |  |  |  |  |  |  |  |  |  |  |  |  |  |  |  |  |  |  |  | T21G5.2 |  |
|  |  |  |  |  |  |  |  |  |  |  |  |  |  |  |  |  |  |  |  |  |  |  |  |  |  |  |  |  |  |  |  |  |  |  |  |  |  | *prk-2* | Pim (mammalian oncogene) Related Kinase |
|  |  |  |  |  |  |  |  |  |  |  |  |  |  |  |  |  |  |  |  |  |  |  |  |  |  |  |  |  |  |  |  |  |  |  |  |  |  | Y47D3B.3 |  |
|  |  |  |  |  |  |  |  |  |  |  |  |  |  |  |  |  |  |  |  |  |  |  |  |  |  |  |  |  |  |  |  |  |  |  |  |  |  | B0034.7 |  |
|  |  |  |  |  |  |  |  |  |  |  |  |  |  |  |  |  |  |  |  |  |  |  |  |  |  |  |  |  |  |  |  |  |  |  |  |  |  | F26E4.3 |  |
|  |  |  |  |  |  |  |  |  |  |  |  |  |  |  |  |  |  |  |  |  |  |  |  |  |  |  |  |  |  |  |  |  |  |  |  |  |  | F49E12.12 |  |
|  |  |  |  |  |  |  |  |  |  |  |  |  |  |  |  |  |  |  |  |  |  |  |  |  |  |  |  |  |  |  |  |  |  |  |  |  |  | *ptr-6* | PaTched Related family |
|  |  |  |  |  |  |  |  |  |  |  |  |  |  |  |  |  |  |  |  |  |  |  |  |  |  |  |  |  |  |  |  |  |  |  |  |  |  | T26C5.2 |  |
|  |  |  |  |  |  |  |  |  |  |  |  |  |  |  |  |  |  |  |  |  |  |  |  |  |  |  |  |  |  |  |  |  |  |  |  |  |  | Y39C12A.1 |  |
|  |  |  |  |  |  |  |  |  |  |  |  |  |  |  |  |  |  |  |  |  |  |  |  |  |  |  |  |  |  |  |  |  |  |  |  |  |  | Y42H9AR.5 |  |
|  |  |  |  |  |  |  |  |  |  |  |  |  |  |  |  |  |  |  |  |  |  |  |  |  |  |  |  |  |  |  |  |  |  |  |  |  |  | *nstp-9* | Nucleotide Sugar TransPorter family |
|  |  |  |  |  |  |  |  |  |  |  |  |  |  |  |  |  |  |  |  |  |  |  |  |  |  |  |  |  |  |  |  |  |  |  |  |  |  | *fipr-21* | FIP (Fungus-Induced Protein) Related |
|  |  |  |  |  |  |  |  |  |  |  |  |  |  |  |  |  |  |  |  |  |  |  |  |  |  |  |  |  |  |  |  |  |  |  |  |  |  | C06C3.10 |  |
|  |  |  |  |  |  |  |  |  |  |  |  |  |  |  |  |  |  |  |  |  |  |  |  |  |  |  |  |  |  |  |  |  |  |  |  |  |  | *nlp-29* | Neuropeptide-Like Protein |
|  |  |  |  |  |  |  |  |  |  |  |  |  |  |  |  |  |  |  |  |  |  |  |  |  |  |  |  |  |  |  |  |  |  |  |  |  |  | ZC434.3 |  |
|  |  |  |  |  |  |  |  |  |  |  |  |  |  |  |  |  |  |  |  |  |  |  |  |  |  |  |  |  |  |  |  |  |  |  |  |  |  | C33B4.2 |  |
|  |  |  |  |  |  |  |  |  |  |  |  |  |  |  |  |  |  |  |  |  |  |  |  |  |  |  |  |  |  |  |  |  |  |  |  |  |  | C23H4.8 |  |
|  |  |  |  |  |  |  |  |  |  |  |  |  |  |  |  |  |  |  |  |  |  |  |  |  |  |  |  |  |  |  |  |  |  |  |  |  |  | W03D2.9 |  |
|  |  |  |  |  |  |  |  |  |  |  |  |  |  |  |  |  |  |  |  |  |  |  |  |  |  |  |  |  |  |  |  |  |  |  |  |  |  | C35A5.3 |  |
|  |  |  |  |  |  |  |  |  |  |  |  |  |  |  |  |  |  |  |  |  |  |  |  |  |  |  |  |  |  |  |  |  |  |  |  |  |  | C26B9.7 |  |
|  |  |  |  |  |  |  |  |  |  |  |  |  |  |  |  |  |  |  |  |  |  |  |  |  |  |  |  |  |  |  |  |  |  |  |  |  |  | C15A11.4 |  |
|  |  |  |  |  |  |  |  |  |  |  |  |  |  |  |  |  |  |  |  |  |  |  |  |  |  |  |  |  |  |  |  |  |  |  |  |  |  | F25E5.2 |  |
|  |  |  |  |  |  |  |  |  |  |  |  |  |  |  |  |  |  |  |  |  |  |  |  |  |  |  |  |  |  |  |  |  |  |  |  |  |  | K02E7.1 |  |
|  |  |  |  |  |  |  |  |  |  |  |  |  |  |  |  |  |  |  |  |  |  |  |  |  |  |  |  |  |  |  |  |  |  |  |  |  |  | T01D1.3 |  |
|  |  |  |  |  |  |  |  |  |  |  |  |  |  |  |  |  |  |  |  |  |  |  |  |  |  |  |  |  |  |  |  |  |  |  |  |  |  | F28C6.5 |  |
|  |  |  |  |  |  |  |  |  |  |  |  |  |  |  |  |  |  |  |  |  |  |  |  |  |  |  |  |  |  |  |  |  |  |  |  |  |  | Y64H9A.2 |  |
|  |  |  |  |  |  |  |  |  |  |  |  |  |  |  |  |  |  |  |  |  |  |  |  |  |  |  |  |  |  |  |  |  |  |  |  |  |  | C14A4.9 |  |
|  |  |  |  |  |  |  |  |  |  |  |  |  |  |  |  |  |  |  |  |  |  |  |  |  |  |  |  |  |  |  |  |  |  |  |  |  |  | Y37A1B.7 |  |
|  |  |  |  |  |  |  |  |  |  |  |  |  |  |  |  |  |  |  |  |  |  |  |  |  |  |  |  |  |  |  |  |  |  |  |  |  |  | C38C6.3 |  |
|  |  |  |  |  |  |  |  |  |  |  |  |  |  |  |  |  |  |  |  |  |  |  |  |  |  |  |  |  |  |  |  |  |  |  |  |  |  | R74.2 |  |
|  |  |  |  |  |  |  |  |  |  |  |  |  |  |  |  |  |  |  |  |  |  |  |  |  |  |  |  |  |  |  |  |  |  |  |  |  |  | T01D1.4 |  |
|  |  |  |  |  |  |  |  |  |  |  |  |  |  |  |  |  |  |  |  |  |  |  |  |  |  |  |  |  |  |  |  |  |  |  |  |  |  | *lgc-22* | Ligand-Gated ion Channel |
|  |  |  |  |  |  |  |  |  |  |  |  |  |  |  |  |  |  |  |  |  |  |  |  |  |  |  |  |  |  |  |  |  |  |  |  |  |  | ZK154.1 |  |
|  |  |  |  |  |  |  |  |  |  |  |  |  |  |  |  |  |  |  |  |  |  |  |  |  |  |  |  |  |  |  |  |  |  |  |  |  |  | ZC123.1 |  |
|  |  |  |  |  |  |  |  |  |  |  |  |  |  |  |  |  |  |  |  |  |  |  |  |  |  |  |  |  |  |  |  |  |  |  |  |  |  | *wrt-4* | WaRThog (hedgehog-like family) |
|  |  |  |  |  |  |  |  |  |  |  |  |  |  |  |  |  |  |  |  |  |  |  |  |  |  |  |  |  |  |  |  |  |  |  |  |  |  | *cut-3* | CUTiclin |
|  |  |  |  |  |  |  |  |  |  |  |  |  |  |  |  |  |  |  |  |  |  |  |  |  |  |  |  |  |  |  |  |  |  |  |  |  |  | K08E7.5 |  |
|  |  |  |  |  |  |  |  |  |  |  |  |  |  |  |  |  |  |  |  |  |  |  |  |  |  |  |  |  |  |  |  |  |  |  |  |  |  | *col-74* | COLlagen |
|  |  |  |  |  |  |  |  |  |  |  |  |  |  |  |  |  |  |  |  |  |  |  |  |  |  |  |  |  |  |  |  |  |  |  |  |  |  | *col-165* | COLlagen |
|  |  |  |  |  |  |  |  |  |  |  |  |  |  |  |  |  |  |  |  |  |  |  |  |  |  |  |  |  |  |  |  |  |  |  |  |  |  | T05H10.3 |  |
|  |  |  |  |  |  |  |  |  |  |  |  |  |  |  |  |  |  |  |  |  |  |  |  |  |  |  |  |  |  |  |  |  |  |  |  |  |  | C49F8.3 |  |
|  |  |  |  |  |  |  |  |  |  |  |  |  |  |  |  |  |  |  |  |  |  |  |  |  |  |  |  |  |  |  |  |  |  |  |  |  |  | F53F1.4 |  |
|  |  |  |  |  |  |  |  |  |  |  |  |  |  |  |  |  |  |  |  |  |  |  |  |  |  |  |  |  |  |  |  |  |  |  |  |  |  | *grl-7* | GRound-Like (grd related) |
|  |  |  |  |  |  |  |  |  |  |  |  |  |  |  |  |  |  |  |  |  |  |  |  |  |  |  |  |  |  |  |  |  |  |  |  |  |  | *grl-15* | GRound-Like (grd related) |
|  |  |  |  |  |  |  |  |  |  |  |  |  |  |  |  |  |  |  |  |  |  |  |  |  |  |  |  |  |  |  |  |  |  |  |  |  |  | *grl-5* | GRound-Like (grd related) |
|  |  |  |  |  |  |  |  |  |  |  |  |  |  |  |  |  |  |  |  |  |  |  |  |  |  |  |  |  |  |  |  |  |  |  |  |  |  | *lpr-7* | LiPocalin-Related protein |
|  |  |  |  |  |  |  |  |  |  |  |  |  |  |  |  |  |  |  |  |  |  |  |  |  |  |  |  |  |  |  |  |  |  |  |  |  |  | F26G1.5 |  |
|  |  |  |  |  |  |  |  |  |  |  |  |  |  |  |  |  |  |  |  |  |  |  |  |  |  |  |  |  |  |  |  |  |  |  |  |  |  | F30A10.2 |  |
|  |  |  |  |  |  |  |  |  |  |  |  |  |  |  |  |  |  |  |  |  |  |  |  |  |  |  |  |  |  |  |  |  |  |  |  |  |  | C50D2.1 |  |
|  |  |  |  |  |  |  |  |  |  |  |  |  |  |  |  |  |  |  |  |  |  |  |  |  |  |  |  |  |  |  |  |  |  |  |  |  |  | F41D9.2 |  |
|  |  |  |  |  |  |  |  |  |  |  |  |  |  |  |  |  |  |  |  |  |  |  |  |  |  |  |  |  |  |  |  |  |  |  |  |  |  | T10G3.3 |  |
|  |  |  |  |  |  |  |  |  |  |  |  |  |  |  |  |  |  |  |  |  |  |  |  |  |  |  |  |  |  |  |  |  |  |  |  |  |  | H13N06.2 |  |
|  |  |  |  |  |  |  |  |  |  |  |  |  |  |  |  |  |  |  |  |  |  |  |  |  |  |  |  |  |  |  |  |  |  |  |  |  |  | *rap-3* | RAP homolog (vertebrate Rap GTPase family) |
|  |  |  |  |  |  |  |  |  |  |  |  |  |  |  |  |  |  |  |  |  |  |  |  |  |  |  |  |  |  |  |  |  |  |  |  |  |  | F44A6.5 |  |
|  |  |  |  |  |  |  |  |  |  |  |  |  |  |  |  |  |  |  |  |  |  |  |  |  |  |  |  |  |  |  |  |  |  |  |  |  |  | F53B1.4 |  |
|  |  |  |  |  |  |  |  |  |  |  |  |  |  |  |  |  |  |  |  |  |  |  |  |  |  |  |  |  |  |  |  |  |  |  |  |  |  | F48D6.4 |  |
|  |  |  |  |  |  |  |  |  |  |  |  |  |  |  |  |  |  |  |  |  |  |  |  |  |  |  |  |  |  |  |  |  |  |  |  |  |  | *clec-180* | C-type LECtin |
|  |  |  |  |  |  |  |  |  |  |  |  |  |  |  |  |  |  |  |  |  |  |  |  |  |  |  |  |  |  |  |  |  |  |  |  |  |  | F18C5.5 |  |
|  |  |  |  |  |  |  |  |  |  |  |  |  |  |  |  |  |  |  |  |  |  |  |  |  |  |  |  |  |  |  |  |  |  |  |  |  |  | C55A6.12 |  |
|  |  |  |  |  |  |  |  |  |  |  |  |  |  |  |  |  |  |  |  |  |  |  |  |  |  |  |  |  |  |  |  |  |  |  |  |  |  | T27A10.6 |  |
|  |  |  |  |  |  |  |  |  |  |  |  |  |  |  |  |  |  |  |  |  |  |  |  |  |  |  |  |  |  |  |  |  |  |  |  |  |  | *wrt-1* | WaRThog (hedgehog-like family) |
|  |  |  |  |  |  |  |  |  |  |  |  |  |  |  |  |  |  |  |  |  |  |  |  |  |  |  |  |  |  |  |  |  |  |  |  |  |  | K08B12.1 |  |
|  |  |  |  |  |  |  |  |  |  |  |  |  |  |  |  |  |  |  |  |  |  |  |  |  |  |  |  |  |  |  |  |  |  |  |  |  |  | *cbn-1* | Calcium BiNding protein homolog |
|  |  |  |  |  |  |  |  |  |  |  |  |  |  |  |  |  |  |  |  |  |  |  |  |  |  |  |  |  |  |  |  |  |  |  |  |  |  | *cut-6* | CUTiclin |
|  |  |  |  |  |  |  |  |  |  |  |  |  |  |  |  |  |  |  |  |  |  |  |  |  |  |  |  |  |  |  |  |  |  |  |  |  |  | Y73E7A.8 |  |
|  |  |  |  |  |  |  |  |  |  |  |  |  |  |  |  |  |  |  |  |  |  |  |  |  |  |  |  |  |  |  |  |  |  |  |  |  |  | ZC449.2 |  |
|  |  |  |  |  |  |  |  |  |  |  |  |  |  |  |  |  |  |  |  |  |  |  |  |  |  |  |  |  |  |  |  |  |  |  |  |  |  | F23H12.5 |  |
|  |  |  |  |  |  |  |  |  |  |  |  |  |  |  |  |  |  |  |  |  |  |  |  |  |  |  |  |  |  |  |  |  |  |  |  |  |  | *osm-8* | OSMotic avoidance abnormal |
|  |  |  |  |  |  |  |  |  |  |  |  |  |  |  |  |  |  |  |  |  |  |  |  |  |  |  |  |  |  |  |  |  |  |  |  |  |  | *glf-1* | GaLactoFuranose synthesis (UGM, UDP-galactopyranose mutase) |
|  |  |  |  |  |  |  |  |  |  |  |  |  |  |  |  |  |  |  |  |  |  |  |  |  |  |  |  |  |  |  |  |  |  |  |  |  |  | *wrt-2* | WaRThog (hedgehog-like family) |
|  |  |  |  |  |  |  |  |  |  |  |  |  |  |  |  |  |  |  |  |  |  |  |  |  |  |  |  |  |  |  |  |  |  |  |  |  |  | T08G5.3 |  |
|  |  |  |  |  |  |  |  |  |  |  |  |  |  |  |  |  |  |  |  |  |  |  |  |  |  |  |  |  |  |  |  |  |  |  |  |  |  | M03B6.3 |  |
|  |  |  |  |  |  |  |  |  |  |  |  |  |  |  |  |  |  |  |  |  |  |  |  |  |  |  |  |  |  |  |  |  |  |  |  |  |  | F22F4.1 |  |
|  |  |  |  |  |  |  |  |  |  |  |  |  |  |  |  |  |  |  |  |  |  |  |  |  |  |  |  |  |  |  |  |  |  |  |  |  |  | *osr-1* | Osmotic Stress Resistant |
|  |  |  |  |  |  |  |  |  |  |  |  |  |  |  |  |  |  |  |  |  |  |  |  |  |  |  |  |  |  |  |  |  |  |  |  |  |  | *mam-3* | MAM (Meprin, A5-protein, PTPmu) domain protein |
|  |  |  |  |  |  |  |  |  |  |  |  |  |  |  |  |  |  |  |  |  |  |  |  |  |  |  |  |  |  |  |  |  |  |  |  |  |  | F15E6.9 |  |
|  |  |  |  |  |  |  |  |  |  |  |  |  |  |  |  |  |  |  |  |  |  |  |  |  |  |  |  |  |  |  |  |  |  |  |  |  |  | *alp-1* | ALP/Enigma encoding |
|  |  |  |  |  |  |  |  |  |  |  |  |  |  |  |  |  |  |  |  |  |  |  |  |  |  |  |  |  |  |  |  |  |  |  |  |  |  | F53H4.3 |  |
|  |  |  |  |  |  |  |  |  |  |  |  |  |  |  |  |  |  |  |  |  |  |  |  |  |  |  |  |  |  |  |  |  |  |  |  |  |  | Y4C6B.3 |  |
|  |  |  |  |  |  |  |  |  |  |  |  |  |  |  |  |  |  |  |  |  |  |  |  |  |  |  |  |  |  |  |  |  |  |  |  |  |  | F25E2.3 |  |
|  |  |  |  |  |  |  |  |  |  |  |  |  |  |  |  |  |  |  |  |  |  |  |  |  |  |  |  |  |  |  |  |  |  |  |  |  |  | F08F3.4 |  |
|  |  |  |  |  |  |  |  |  |  |  |  |  |  |  |  |  |  |  |  |  |  |  |  |  |  |  |  |  |  |  |  |  |  |  |  |  |  | C07A9.5 |  |
|  |  |  |  |  |  |  |  |  |  |  |  |  |  |  |  |  |  |  |  |  |  |  |  |  |  |  |  |  |  |  |  |  |  |  |  |  |  | C01G6.9 |  |
|  |  |  |  |  |  |  |  |  |  |  |  |  |  |  |  |  |  |  |  |  |  |  |  |  |  |  |  |  |  |  |  |  |  |  |  |  |  | *suro-1* | SUPpressor of ROller |
|  |  |  |  |  |  |  |  |  |  |  |  |  |  |  |  |  |  |  |  |  |  |  |  |  |  |  |  |  |  |  |  |  |  |  |  |  |  | Y62E10A.19 |  |
|  |  |  |  |  |  |  |  |  |  |  |  |  |  |  |  |  |  |  |  |  |  |  |  |  |  |  |  |  |  |  |  |  |  |  |  |  |  | T28A11.2 |  |
|  |  |  |  |  |  |  |  |  |  |  |  |  |  |  |  |  |  |  |  |  |  |  |  |  |  |  |  |  |  |  |  |  |  |  |  |  |  | C35A5.6 |  |
|  |  |  |  |  |  |  |  |  |  |  |  |  |  |  |  |  |  |  |  |  |  |  |  |  |  |  |  |  |  |  |  |  |  |  |  |  |  | ZK666.14 |  |
|  |  |  |  |  |  |  |  |  |  |  |  |  |  |  |  |  |  |  |  |  |  |  |  |  |  |  |  |  |  |  |  |  |  |  |  |  |  | F53F4.15 |  |
|  |  |  |  |  |  |  |  |  |  |  |  |  |  |  |  |  |  |  |  |  |  |  |  |  |  |  |  |  |  |  |  |  |  |  |  |  |  | *grl-10* | GRound-Like (grd related) |
|  |  |  |  |  |  |  |  |  |  |  |  |  |  |  |  |  |  |  |  |  |  |  |  |  |  |  |  |  |  |  |  |  |  |  |  |  |  | C17F4.2 |  |
|  |  |  |  |  |  |  |  |  |  |  |  |  |  |  |  |  |  |  |  |  |  |  |  |  |  |  |  |  |  |  |  |  |  |  |  |  |  | C07A9.13 |  |
|  |  |  |  |  |  |  |  |  |  |  |  |  |  |  |  |  |  |  |  |  |  |  |  |  |  |  |  |  |  |  |  |  |  |  |  |  |  | ZK829.3 |  |
|  |  |  |  |  |  |  |  |  |  |  |  |  |  |  |  |  |  |  |  |  |  |  |  |  |  |  |  |  |  |  |  |  |  |  |  |  |  | T22D1.18 |  |
|  |  |  |  |  |  |  |  |  |  |  |  |  |  |  |  |  |  |  |  |  |  |  |  |  |  |  |  |  |  |  |  |  |  |  |  |  |  | Y45F10B.59 |  |
|  |  |  |  |  |  |  |  |  |  |  |  |  |  |  |  |  |  |  |  |  |  |  |  |  |  |  |  |  |  |  |  |  |  |  |  |  |  | F22F7.6 |  |
|  |  |  |  |  |  |  |  |  |  |  |  |  |  |  |  |  |  |  |  |  |  |  |  |  |  |  |  |  |  |  |  |  |  |  |  |  |  | F35G2.5 |  |
|  |  |  |  |  |  |  |  |  |  |  |  |  |  |  |  |  |  |  |  |  |  |  |  |  |  |  |  |  |  |  |  |  |  |  |  |  |  | F13A7.12 |  |
|  |  |  |  |  |  |  |  |  |  |  |  |  |  |  |  |  |  |  |  |  |  |  |  |  |  |  |  |  |  |  |  |  |  |  |  |  |  | F17B5.6 |  |
|  |  |  |  |  |  |  |  |  |  |  |  |  |  |  |  |  |  |  |  |  |  |  |  |  |  |  |  |  |  |  |  |  |  |  |  |  |  | *lon-3* | LONg |
|  |  |  |  |  |  |  |  |  |  |  |  |  |  |  |  |  |  |  |  |  |  |  |  |  |  |  |  |  |  |  |  |  |  |  |  |  |  | F18A11.2 |  |
|  |  |  |  |  |  |  |  |  |  |  |  |  |  |  |  |  |  |  |  |  |  |  |  |  |  |  |  |  |  |  |  |  |  |  |  |  |  | *nstp-8* | Nucleotide Sugar TransPorter family |
|  |  |  |  |  |  |  |  |  |  |  |  |  |  |  |  |  |  |  |  |  |  |  |  |  |  |  |  |  |  |  |  |  |  |  |  |  |  | F47B8.5 |  |
|  |  |  |  |  |  |  |  |  |  |  |  |  |  |  |  |  |  |  |  |  |  |  |  |  |  |  |  |  |  |  |  |  |  |  |  |  |  | F49D11.6 |  |
|  |  |  |  |  |  |  |  |  |  |  |  |  |  |  |  |  |  |  |  |  |  |  |  |  |  |  |  |  |  |  |  |  |  |  |  |  |  | F58E6.3 |  |
|  |  |  |  |  |  |  |  |  |  |  |  |  |  |  |  |  |  |  |  |  |  |  |  |  |  |  |  |  |  |  |  |  |  |  |  |  |  | K01D12.1 |  |
|  |  |  |  |  |  |  |  |  |  |  |  |  |  |  |  |  |  |  |  |  |  |  |  |  |  |  |  |  |  |  |  |  |  |  |  |  |  | F11A5.5 |  |
|  |  |  |  |  |  |  |  |  |  |  |  |  |  |  |  |  |  |  |  |  |  |  |  |  |  |  |  |  |  |  |  |  |  |  |  |  |  | F21H7.10 |  |
|  |  |  |  |  |  |  |  |  |  |  |  |  |  |  |  |  |  |  |  |  |  |  |  |  |  |  |  |  |  |  |  |  |  |  |  |  |  | F15H10.8 |  |
|  |  |  |  |  |  |  |  |  |  |  |  |  |  |  |  |  |  |  |  |  |  |  |  |  |  |  |  |  |  |  |  |  |  |  |  |  |  | F37A4.3 |  |
|  |  |  |  |  |  |  |  |  |  |  |  |  |  |  |  |  |  |  |  |  |  |  |  |  |  |  |  |  |  |  |  |  |  |  |  |  |  | W08G11.1 |  |
|  |  |  |  |  |  |  |  |  |  |  |  |  |  |  |  |  |  |  |  |  |  |  |  |  |  |  |  |  |  |  |  |  |  |  |  |  |  | *oac-51* | O-ACyltransferase homolog |
|  |  |  |  |  |  |  |  |  |  |  |  |  |  |  |  |  |  |  |  |  |  |  |  |  |  |  |  |  |  |  |  |  |  |  |  |  |  | F36F12.1 |  |
|  |  |  |  |  |  |  |  |  |  |  |  |  |  |  |  |  |  |  |  |  |  |  |  |  |  |  |  |  |  |  |  |  |  |  |  |  |  | F30A10.13 |  |
|  |  |  |  |  |  |  |  |  |  |  |  |  |  |  |  |  |  |  |  |  |  |  |  |  |  |  |  |  |  |  |  |  |  |  |  |  |  | T07G12.3 |  |
|  |  |  |  |  |  |  |  |  |  |  |  |  |  |  |  |  |  |  |  |  |  |  |  |  |  |  |  |  |  |  |  |  |  |  |  |  |  | R09B5.11 |  |
|  |  |  |  |  |  |  |  |  |  |  |  |  |  |  |  |  |  |  |  |  |  |  |  |  |  |  |  |  |  |  |  |  |  |  |  |  |  | R08C7.4 |  |
|  |  |  |  |  |  |  |  |  |  |  |  |  |  |  |  |  |  |  |  |  |  |  |  |  |  |  |  |  |  |  |  |  |  |  |  |  |  | T06A1.1 |  |
|  |  |  |  |  |  |  |  |  |  |  |  |  |  |  |  |  |  |  |  |  |  |  |  |  |  |  |  |  |  |  |  |  |  |  |  |  |  | *cut-5* | CUTiclin |
|  |  |  |  |  |  |  |  |  |  |  |  |  |  |  |  |  |  |  |  |  |  |  |  |  |  |  |  |  |  |  |  |  |  |  |  |  |  | R05G9R.1 |  |
|  |  |  |  |  |  |  |  |  |  |  |  |  |  |  |  |  |  |  |  |  |  |  |  |  |  |  |  |  |  |  |  |  |  |  |  |  |  | T03G6.1 |  |
|  |  |  |  |  |  |  |  |  |  |  |  |  |  |  |  |  |  |  |  |  |  |  |  |  |  |  |  |  |  |  |  |  |  |  |  |  |  | D1014.5 |  |
|  |  |  |  |  |  |  |  |  |  |  |  |  |  |  |  |  |  |  |  |  |  |  |  |  |  |  |  |  |  |  |  |  |  |  |  |  |  | *nstp-3* | Nucleotide Sugar TransPorter family |
|  |  |  |  |  |  |  |  |  |  |  |  |  |  |  |  |  |  |  |  |  |  |  |  |  |  |  |  |  |  |  |  |  |  |  |  |  |  | F29C6.1 |  |
|  |  |  |  |  |  |  |  |  |  |  |  |  |  |  |  |  |  |  |  |  |  |  |  |  |  |  |  |  |  |  |  |  |  |  |  |  |  | B0252.3 |  |
|  |  |  |  |  |  |  |  |  |  |  |  |  |  |  |  |  |  |  |  |  |  |  |  |  |  |  |  |  |  |  |  |  |  |  |  |  |  | F38A6.5 |  |
|  |  |  |  |  |  |  |  |  |  |  |  |  |  |  |  |  |  |  |  |  |  |  |  |  |  |  |  |  |  |  |  |  |  |  |  |  |  | C54F6.3 |  |
|  |  |  |  |  |  |  |  |  |  |  |  |  |  |  |  |  |  |  |  |  |  |  |  |  |  |  |  |  |  |  |  |  |  |  |  |  |  | Y75B12B.11 |  |
|  |  |  |  |  |  |  |  |  |  |  |  |  |  |  |  |  |  |  |  |  |  |  |  |  |  |  |  |  |  |  |  |  |  |  |  |  |  | *ghi-1* | Glycoprotein Hormone (FLR-2) Interacting protein |
|  |  |  |  |  |  |  |  |  |  |  |  |  |  |  |  |  |  |  |  |  |  |  |  |  |  |  |  |  |  |  |  |  |  |  |  |  |  | *nekl-3* | NEK (NEver in mitosis Kinase) Like |
|  |  |  |  |  |  |  |  |  |  |  |  |  |  |  |  |  |  |  |  |  |  |  |  |  |  |  |  |  |  |  |  |  |  |  |  |  |  | F45D3.3 |  |
|  |  |  |  |  |  |  |  |  |  |  |  |  |  |  |  |  |  |  |  |  |  |  |  |  |  |  |  |  |  |  |  |  |  |  |  |  |  | *nhr-260* | Nuclear Hormone Receptor family |
|  |  |  |  |  |  |  |  |  |  |  |  |  |  |  |  |  |  |  |  |  |  |  |  |  |  |  |  |  |  |  |  |  |  |  |  |  |  | F48F7.3 |  |
|  |  |  |  |  |  |  |  |  |  |  |  |  |  |  |  |  |  |  |  |  |  |  |  |  |  |  |  |  |  |  |  |  |  |  |  |  |  | *dpy-4* | DumPY: shorter than wild-type |
|  |  |  |  |  |  |  |  |  |  |  |  |  |  |  |  |  |  |  |  |  |  |  |  |  |  |  |  |  |  |  |  |  |  |  |  |  |  | *col-117* | COLlagen |
|  |  |  |  |  |  |  |  |  |  |  |  |  |  |  |  |  |  |  |  |  |  |  |  |  |  |  |  |  |  |  |  |  |  |  |  |  |  | *col-157* | COLlagen |
|  |  |  |  |  |  |  |  |  |  |  |  |  |  |  |  |  |  |  |  |  |  |  |  |  |  |  |  |  |  |  |  |  |  |  |  |  |  | *ifa-3* | Intermediate Filament, A |
|  |  |  |  |  |  |  |  |  |  |  |  |  |  |  |  |  |  |  |  |  |  |  |  |  |  |  |  |  |  |  |  |  |  |  |  |  |  | Y41D4B.6 |  |
|  |  |  |  |  |  |  |  |  |  |  |  |  |  |  |  |  |  |  |  |  |  |  |  |  |  |  |  |  |  |  |  |  |  |  |  |  |  | *dsl-6* | Delta/Serrate/Lag-2 domain |
|  |  |  |  |  |  |  |  |  |  |  |  |  |  |  |  |  |  |  |  |  |  |  |  |  |  |  |  |  |  |  |  |  |  |  |  |  |  | *dpy-2* | DumPY: shorter than wild-type |
|  |  |  |  |  |  |  |  |  |  |  |  |  |  |  |  |  |  |  |  |  |  |  |  |  |  |  |  |  |  |  |  |  |  |  |  |  |  | C14F11.6 |  |
|  |  |  |  |  |  |  |  |  |  |  |  |  |  |  |  |  |  |  |  |  |  |  |  |  |  |  |  |  |  |  |  |  |  |  |  |  |  | F32D1.11 |  |
|  |  |  |  |  |  |  |  |  |  |  |  |  |  |  |  |  |  |  |  |  |  |  |  |  |  |  |  |  |  |  |  |  |  |  |  |  |  | F25E5.8 |  |
|  |  |  |  |  |  |  |  |  |  |  |  |  |  |  |  |  |  |  |  |  |  |  |  |  |  |  |  |  |  |  |  |  |  |  |  |  |  | T01D1.8 |  |
|  |  |  |  |  |  |  |  |  |  |  |  |  |  |  |  |  |  |  |  |  |  |  |  |  |  |  |  |  |  |  |  |  |  |  |  |  |  | *grl-24* | GRound-Like (grd related) |
|  |  |  |  |  |  |  |  |  |  |  |  |  |  |  |  |  |  |  |  |  |  |  |  |  |  |  |  |  |  |  |  |  |  |  |  |  |  | F11E6.9 |  |
|  |  |  |  |  |  |  |  |  |  |  |  |  |  |  |  |  |  |  |  |  |  |  |  |  |  |  |  |  |  |  |  |  |  |  |  |  |  | F41E6.8 |  |
|  |  |  |  |  |  |  |  |  |  |  |  |  |  |  |  |  |  |  |  |  |  |  |  |  |  |  |  |  |  |  |  |  |  |  |  |  |  | C44E12.1 |  |
|  |  |  |  |  |  |  |  |  |  |  |  |  |  |  |  |  |  |  |  |  |  |  |  |  |  |  |  |  |  |  |  |  |  |  |  |  |  | *srm-1* | Serpentine Receptor, class M |
|  |  |  |  |  |  |  |  |  |  |  |  |  |  |  |  |  |  |  |  |  |  |  |  |  |  |  |  |  |  |  |  |  |  |  |  |  |  | B0205.4 |  |
|  |  |  |  |  |  |  |  |  |  |  |  |  |  |  |  |  |  |  |  |  |  |  |  |  |  |  |  |  |  |  |  |  |  |  |  |  |  | *chw-1* | CHp/Wrch Rho-like protein homolog |
|  |  |  |  |  |  |  |  |  |  |  |  |  |  |  |  |  |  |  |  |  |  |  |  |  |  |  |  |  |  |  |  |  |  |  |  |  |  | W03F11.5 |  |
|  |  |  |  |  |  |  |  |  |  |  |  |  |  |  |  |  |  |  |  |  |  |  |  |  |  |  |  |  |  |  |  |  |  |  |  |  |  | F25B4.8 |  |
|  |  |  |  |  |  |  |  |  |  |  |  |  |  |  |  |  |  |  |  |  |  |  |  |  |  |  |  |  |  |  |  |  |  |  |  |  |  | C26F1.1 |  |
|  |  |  |  |  |  |  |  |  |  |  |  |  |  |  |  |  |  |  |  |  |  |  |  |  |  |  |  |  |  |  |  |  |  |  |  |  |  | F40A3.2 |  |
|  |  |  |  |  |  |  |  |  |  |  |  |  |  |  |  |  |  |  |  |  |  |  |  |  |  |  |  |  |  |  |  |  |  |  |  |  |  | C35A5.11 |  |
|  |  |  |  |  |  |  |  |  |  |  |  |  |  |  |  |  |  |  |  |  |  |  |  |  |  |  |  |  |  |  |  |  |  |  |  |  |  | Y54G2A.37 |  |
|  |  |  |  |  |  |  |  |  |  |  |  |  |  |  |  |  |  |  |  |  |  |  |  |  |  |  |  |  |  |  |  |  |  |  |  |  |  | T13C5.3 |  |
|  |  |  |  |  |  |  |  |  |  |  |  |  |  |  |  |  |  |  |  |  |  |  |  |  |  |  |  |  |  |  |  |  |  |  |  |  |  | F46F11.7 |  |
|  |  |  |  |  |  |  |  |  |  |  |  |  |  |  |  |  |  |  |  |  |  |  |  |  |  |  |  |  |  |  |  |  |  |  |  |  |  | F15B9.8 |  |
|  |  |  |  |  |  |  |  |  |  |  |  |  |  |  |  |  |  |  |  |  |  |  |  |  |  |  |  |  |  |  |  |  |  |  |  |  |  | W03D8.11 |  |
|  |  |  |  |  |  |  |  |  |  |  |  |  |  |  |  |  |  |  |  |  |  |  |  |  |  |  |  |  |  |  |  |  |  |  |  |  |  | C53A5.2 |  |
|  |  |  |  |  |  |  |  |  |  |  |  |  |  |  |  |  |  |  |  |  |  |  |  |  |  |  |  |  |  |  |  |  |  |  |  |  |  | C06C3.4 |  |
|  |  |  |  |  |  |  |  |  |  |  |  |  |  |  |  |  |  |  |  |  |  |  |  |  |  |  |  |  |  |  |  |  |  |  |  |  |  | M03F8.1 |  |
|  |  |  |  |  |  |  |  |  |  |  |  |  |  |  |  |  |  |  |  |  |  |  |  |  |  |  |  |  |  |  |  |  |  |  |  |  |  | T25B9.1 |  |
|  |  |  |  |  |  |  |  |  |  |  |  |  |  |  |  |  |  |  |  |  |  |  |  |  |  |  |  |  |  |  |  |  |  |  |  |  |  | *elo-4* | fatty acid ELOngation |
|  |  |  |  |  |  |  |  |  |  |  |  |  |  |  |  |  |  |  |  |  |  |  |  |  |  |  |  |  |  |  |  |  |  |  |  |  |  | F32D8.3 |  |
|  |  |  |  |  |  |  |  |  |  |  |  |  |  |  |  |  |  |  |  |  |  |  |  |  |  |  |  |  |  |  |  |  |  |  |  |  |  | *sqt-3* | SQuaT |
|  |  |  |  |  |  |  |  |  |  |  |  |  |  |  |  |  |  |  |  |  |  |  |  |  |  |  |  |  |  |  |  |  |  |  |  |  |  | *dpy-3* | DumPY: shorter than wild-type |
|  |  |  |  |  |  |  |  |  |  |  |  |  |  |  |  |  |  |  |  |  |  |  |  |  |  |  |  |  |  |  |  |  |  |  |  |  |  | *dpy-14* | DumPY: shorter than wild-type |
|  |  |  |  |  |  |  |  |  |  |  |  |  |  |  |  |  |  |  |  |  |  |  |  |  |  |  |  |  |  |  |  |  |  |  |  |  |  | *dpy-17* | DumPY: shorter than wild-type |
|  |  |  |  |  |  |  |  |  |  |  |  |  |  |  |  |  |  |  |  |  |  |  |  |  |  |  |  |  |  |  |  |  |  |  |  |  |  | F31C3.6 |  |
|  |  |  |  |  |  |  |  |  |  |  |  |  |  |  |  |  |  |  |  |  |  |  |  |  |  |  |  |  |  |  |  |  |  |  |  |  |  | *dao-2* | Dauer or Aging adult Overexpression |
|  |  |  |  |  |  |  |  |  |  |  |  |  |  |  |  |  |  |  |  |  |  |  |  |  |  |  |  |  |  |  |  |  |  |  |  |  |  | K02E10.4 |  |
|  |  |  |  |  |  |  |  |  |  |  |  |  |  |  |  |  |  |  |  |  |  |  |  |  |  |  |  |  |  |  |  |  |  |  |  |  |  | T19C4.1 |  |
|  |  |  |  |  |  |  |  |  |  |  |  |  |  |  |  |  |  |  |  |  |  |  |  |  |  |  |  |  |  |  |  |  |  |  |  |  |  | F43D9.1 |  |
|  |  |  |  |  |  |  |  |  |  |  |  |  |  |  |  |  |  |  |  |  |  |  |  |  |  |  |  |  |  |  |  |  |  |  |  |  |  | *col-121* | COLlagen |
|  |  |  |  |  |  |  |  |  |  |  |  |  |  |  |  |  |  |  |  |  |  |  |  |  |  |  |  |  |  |  |  |  |  |  |  |  |  | *dpy-7* | DumPY: shorter than wild-type |
|  |  |  |  |  |  |  |  |  |  |  |  |  |  |  |  |  |  |  |  |  |  |  |  |  |  |  |  |  |  |  |  |  |  |  |  |  |  | *cysl-2* | CYsteine Synthase Like |
|  |  |  |  |  |  |  |  |  |  |  |  |  |  |  |  |  |  |  |  |  |  |  |  |  |  |  |  |  |  |  |  |  |  |  |  |  |  | R11F4.1 |  |
|  |  |  |  |  |  |  |  |  |  |  |  |  |  |  |  |  |  |  |  |  |  |  |  |  |  |  |  |  |  |  |  |  |  |  |  |  |  | *nhr-114* | Nuclear Hormone Receptor family |
|  |  |  |  |  |  |  |  |  |  |  |  |  |  |  |  |  |  |  |  |  |  |  |  |  |  |  |  |  |  |  |  |  |  |  |  |  |  | Y47G6A.19 |  |
|  |  |  |  |  |  |  |  |  |  |  |  |  |  |  |  |  |  |  |  |  |  |  |  |  |  |  |  |  |  |  |  |  |  |  |  |  |  | Y50D7A.13 |  |
|  |  |  |  |  |  |  |  |  |  |  |  |  |  |  |  |  |  |  |  |  |  |  |  |  |  |  |  |  |  |  |  |  |  |  |  |  |  | F41F3.8 |  |
|  |  |  |  |  |  |  |  |  |  |  |  |  |  |  |  |  |  |  |  |  |  |  |  |  |  |  |  |  |  |  |  |  |  |  |  |  |  | Y71G12B.17 |  |
|  |  |  |  |  |  |  |  |  |  |  |  |  |  |  |  |  |  |  |  |  |  |  |  |  |  |  |  |  |  |  |  |  |  |  |  |  |  | *mltn-12* | MLt-TeN (mlt-10) related |
|  |  |  |  |  |  |  |  |  |  |  |  |  |  |  |  |  |  |  |  |  |  |  |  |  |  |  |  |  |  |  |  |  |  |  |  |  |  | K08B5.1 |  |
|  |  |  |  |  |  |  |  |  |  |  |  |  |  |  |  |  |  |  |  |  |  |  |  |  |  |  |  |  |  |  |  |  |  |  |  |  |  | *mltn-9* | MLt-TeN (mlt-10) related |
|  |  |  |  |  |  |  |  |  |  |  |  |  |  |  |  |  |  |  |  |  |  |  |  |  |  |  |  |  |  |  |  |  |  |  |  |  |  | *tag-297* | Temporarily Assigned Gene name |
|  |  |  |  |  |  |  |  |  |  |  |  |  |  |  |  |  |  |  |  |  |  |  |  |  |  |  |  |  |  |  |  |  |  |  |  |  |  | Y39A3CR.5 |  |
|  |  |  |  |  |  |  |  |  |  |  |  |  |  |  |  |  |  |  |  |  |  |  |  |  |  |  |  |  |  |  |  |  |  |  |  |  |  | *hlh-33* | Helix Loop Helix |
|  |  |  |  |  |  |  |  |  |  |  |  |  |  |  |  |  |  |  |  |  |  |  |  |  |  |  |  |  |  |  |  |  |  |  |  |  |  | *bus-19* | Bacterially Un-Swollen (M. nematophilum resistant) |
|  |  |  |  |  |  |  |  |  |  |  |  |  |  |  |  |  |  |  |  |  |  |  |  |  |  |  |  |  |  |  |  |  |  |  |  |  |  | F46C8.8 |  |
|  |  |  |  |  |  |  |  |  |  |  |  |  |  |  |  |  |  |  |  |  |  |  |  |  |  |  |  |  |  |  |  |  |  |  |  |  |  | F09F9.2 |  |
|  |  |  |  |  |  |  |  |  |  |  |  |  |  |  |  |  |  |  |  |  |  |  |  |  |  |  |  |  |  |  |  |  |  |  |  |  |  | *arrd-10* | ARRestin Domain protein |
|  |  |  |  |  |  |  |  |  |  |  |  |  |  |  |  |  |  |  |  |  |  |  |  |  |  |  |  |  |  |  |  |  |  |  |  |  |  | *grd-5* | GRounDhog (hedgehog-like family) |
|  |  |  |  |  |  |  |  |  |  |  |  |  |  |  |  |  |  |  |  |  |  |  |  |  |  |  |  |  |  |  |  |  |  |  |  |  |  | *gfrp-1* | GTP cyclohydrolase I Feedback Regulatory Protein homolog |
|  |  |  |  |  |  |  |  |  |  |  |  |  |  |  |  |  |  |  |  |  |  |  |  |  |  |  |  |  |  |  |  |  |  |  |  |  |  | Y43F8B.1 |  |
|  |  |  |  |  |  |  |  |  |  |  |  |  |  |  |  |  |  |  |  |  |  |  |  |  |  |  |  |  |  |  |  |  |  |  |  |  |  | *dpy-1* | DumPY: shorter than wild-type |
|  |  |  |  |  |  |  |  |  |  |  |  |  |  |  |  |  |  |  |  |  |  |  |  |  |  |  |  |  |  |  |  |  |  |  |  |  |  | *elp-1* | Emap-Like Protein |
|  |  |  |  |  |  |  |  |  |  |  |  |  |  |  |  |  |  |  |  |  |  |  |  |  |  |  |  |  |  |  |  |  |  |  |  |  |  | C27H5.2 |  |
|  |  |  |  |  |  |  |  |  |  |  |  |  |  |  |  |  |  |  |  |  |  |  |  |  |  |  |  |  |  |  |  |  |  |  |  |  |  | *catp-1* | Cation transporting ATPase |
|  |  |  |  |  |  |  |  |  |  |  |  |  |  |  |  |  |  |  |  |  |  |  |  |  |  |  |  |  |  |  |  |  |  |  |  |  |  | *sams-1* | S-Adenosyl Methionine Synthetase |
|  |  |  |  |  |  |  |  |  |  |  |  |  |  |  |  |  |  |  |  |  |  |  |  |  |  |  |  |  |  |  |  |  |  |  |  |  |  | C42D4.3 |  |
|  |  |  |  |  |  |  |  |  |  |  |  |  |  |  |  |  |  |  |  |  |  |  |  |  |  |  |  |  |  |  |  |  |  |  |  |  |  | *grl-4* | GRound-Like (grd related) |
|  |  |  |  |  |  |  |  |  |  |  |  |  |  |  |  |  |  |  |  |  |  |  |  |  |  |  |  |  |  |  |  |  |  |  |  |  |  | *wrt-9* | WaRThog (hedgehog-like family) |
|  |  |  |  |  |  |  |  |  |  |  |  |  |  |  |  |  |  |  |  |  |  |  |  |  |  |  |  |  |  |  |  |  |  |  |  |  |  | F18E9.3 |  |
|  |  |  |  |  |  |  |  |  |  |  |  |  |  |  |  |  |  |  |  |  |  |  |  |  |  |  |  |  |  |  |  |  |  |  |  |  |  | F01G10.10 |  |
|  |  |  |  |  |  |  |  |  |  |  |  |  |  |  |  |  |  |  |  |  |  |  |  |  |  |  |  |  |  |  |  |  |  |  |  |  |  | F41E6.12 |  |
|  |  |  |  |  |  |  |  |  |  |  |  |  |  |  |  |  |  |  |  |  |  |  |  |  |  |  |  |  |  |  |  |  |  |  |  |  |  | *col-76* | COLlagen |
|  |  |  |  |  |  |  |  |  |  |  |  |  |  |  |  |  |  |  |  |  |  |  |  |  |  |  |  |  |  |  |  |  |  |  |  |  |  | *tba-4* | TuBulin, Alpha |
|  |  |  |  |  |  |  |  |  |  |  |  |  |  |  |  |  |  |  |  |  |  |  |  |  |  |  |  |  |  |  |  |  |  |  |  |  |  | *rol-3* | ROLler: helically twisted, animals roll when moving |
|  |  |  |  |  |  |  |  |  |  |  |  |  |  |  |  |  |  |  |  |  |  |  |  |  |  |  |  |  |  |  |  |  |  |  |  |  |  | K09E4.4 |  |
|  |  |  |  |  |  |  |  |  |  |  |  |  |  |  |  |  |  |  |  |  |  |  |  |  |  |  |  |  |  |  |  |  |  |  |  |  |  | C16C8.2 |  |
|  |  |  |  |  |  |  |  |  |  |  |  |  |  |  |  |  |  |  |  |  |  |  |  |  |  |  |  |  |  |  |  |  |  |  |  |  |  | *pah-1* | PhenylAlanine Hydroxylase |
|  |  |  |  |  |  |  |  |  |  |  |  |  |  |  |  |  |  |  |  |  |  |  |  |  |  |  |  |  |  |  |  |  |  |  |  |  |  | Y81G3A.4 |  |
|  |  |  |  |  |  |  |  |  |  |  |  |  |  |  |  |  |  |  |  |  |  |  |  |  |  |  |  |  |  |  |  |  |  |  |  |  |  | *swt-5* | SWEET sugar transporter family |
|  |  |  |  |  |  |  |  |  |  |  |  |  |  |  |  |  |  |  |  |  |  |  |  |  |  |  |  |  |  |  |  |  |  |  |  |  |  | Y38A10A.2 |  |
|  |  |  |  |  |  |  |  |  |  |  |  |  |  |  |  |  |  |  |  |  |  |  |  |  |  |  |  |  |  |  |  |  |  |  |  |  |  | T16G1.2 |  |
|  |  |  |  |  |  |  |  |  |  |  |  |  |  |  |  |  |  |  |  |  |  |  |  |  |  |  |  |  |  |  |  |  |  |  |  |  |  | *lips-7* | LIPaSe related |
|  |  |  |  |  |  |  |  |  |  |  |  |  |  |  |  |  |  |  |  |  |  |  |  |  |  |  |  |  |  |  |  |  |  |  |  |  |  | R02E4.3 |  |
|  |  |  |  |  |  |  |  |  |  |  |  |  |  |  |  |  |  |  |  |  |  |  |  |  |  |  |  |  |  |  |  |  |  |  |  |  |  | F13D12.9 |  |
|  |  |  |  |  |  |  |  |  |  |  |  |  |  |  |  |  |  |  |  |  |  |  |  |  |  |  |  |  |  |  |  |  |  |  |  |  |  | *abu-13* | Activated in Blocked Unfolded protein response |
|  |  |  |  |  |  |  |  |  |  |  |  |  |  |  |  |  |  |  |  |  |  |  |  |  |  |  |  |  |  |  |  |  |  |  |  |  |  | T19A5.3 |  |
|  |  |  |  |  |  |  |  |  |  |  |  |  |  |  |  |  |  |  |  |  |  |  |  |  |  |  |  |  |  |  |  |  |  |  |  |  |  | *act-1* | ACTin |
|  |  |  |  |  |  |  |  |  |  |  |  |  |  |  |  |  |  |  |  |  |  |  |  |  |  |  |  |  |  |  |  |  |  |  |  |  |  | Y65B4BL.6 |  |
|  |  |  |  |  |  |  |  |  |  |  |  |  |  |  |  |  |  |  |  |  |  |  |  |  |  |  |  |  |  |  |  |  |  |  |  |  |  | Y50E8A.1 |  |
|  |  |  |  |  |  |  |  |  |  |  |  |  |  |  |  |  |  |  |  |  |  |  |  |  |  |  |  |  |  |  |  |  |  |  |  |  |  | *qua-1* | QUAhog (hedgehog related) |
|  |  |  |  |  |  |  |  |  |  |  |  |  |  |  |  |  |  |  |  |  |  |  |  |  |  |  |  |  |  |  |  |  |  |  |  |  |  | *grl-16* | GRound-Like (grd related) |
|  |  |  |  |  |  |  |  |  |  |  |  |  |  |  |  |  |  |  |  |  |  |  |  |  |  |  |  |  |  |  |  |  |  |  |  |  |  | F10D11.6 |  |
|  |  |  |  |  |  |  |  |  |  |  |  |  |  |  |  |  |  |  |  |  |  |  |  |  |  |  |  |  |  |  |  |  |  |  |  |  |  | F32D8.10 |  |
|  |  |  |  |  |  |  |  |  |  |  |  |  |  |  |  |  |  |  |  |  |  |  |  |  |  |  |  |  |  |  |  |  |  |  |  |  |  | *mab-7* | Male ABnormal |
|  |  |  |  |  |  |  |  |  |  |  |  |  |  |  |  |  |  |  |  |  |  |  |  |  |  |  |  |  |  |  |  |  |  |  |  |  |  | *pqn-72* | Prion-like-(Q/N-rich)-domain-bearing protein |
|  |  |  |  |  |  |  |  |  |  |  |  |  |  |  |  |  |  |  |  |  |  |  |  |  |  |  |  |  |  |  |  |  |  |  |  |  |  | *ncx-1* | Na/Ca eXchangers |
|  |  |  |  |  |  |  |  |  |  |  |  |  |  |  |  |  |  |  |  |  |  |  |  |  |  |  |  |  |  |  |  |  |  |  |  |  |  | C55C3.1 |  |
|  |  |  |  |  |  |  |  |  |  |  |  |  |  |  |  |  |  |  |  |  |  |  |  |  |  |  |  |  |  |  |  |  |  |  |  |  |  | B0303.11 |  |
|  |  |  |  |  |  |  |  |  |  |  |  |  |  |  |  |  |  |  |  |  |  |  |  |  |  |  |  |  |  |  |  |  |  |  |  |  |  | T28C12.4 |  |
|  |  |  |  |  |  |  |  |  |  |  |  |  |  |  |  |  |  |  |  |  |  |  |  |  |  |  |  |  |  |  |  |  |  |  |  |  |  | Y57G11A.4 |  |
|  |  |  |  |  |  |  |  |  |  |  |  |  |  |  |  |  |  |  |  |  |  |  |  |  |  |  |  |  |  |  |  |  |  |  |  |  |  | Y53F4B.25 |  |
|  |  |  |  |  |  |  |  |  |  |  |  |  |  |  |  |  |  |  |  |  |  |  |  |  |  |  |  |  |  |  |  |  |  |  |  |  |  | *ptr-18* | PaTched Related family |
|  |  |  |  |  |  |  |  |  |  |  |  |  |  |  |  |  |  |  |  |  |  |  |  |  |  |  |  |  |  |  |  |  |  |  |  |  |  | *moe-3* | see oma |
|  |  |  |  |  |  |  |  |  |  |  |  |  |  |  |  |  |  |  |  |  |  |  |  |  |  |  |  |  |  |  |  |  |  |  |  |  |  | *lips-3* | LIPaSe related |
|  |  |  |  |  |  |  |  |  |  |  |  |  |  |  |  |  |  |  |  |  |  |  |  |  |  |  |  |  |  |  |  |  |  |  |  |  |  | ZK180.5 |  |
|  |  |  |  |  |  |  |  |  |  |  |  |  |  |  |  |  |  |  |  |  |  |  |  |  |  |  |  |  |  |  |  |  |  |  |  |  |  | *col-10* | COLlagen |
|  |  |  |  |  |  |  |  |  |  |  |  |  |  |  |  |  |  |  |  |  |  |  |  |  |  |  |  |  |  |  |  |  |  |  |  |  |  | C46A5.4 |  |
|  |  |  |  |  |  |  |  |  |  |  |  |  |  |  |  |  |  |  |  |  |  |  |  |  |  |  |  |  |  |  |  |  |  |  |  |  |  | T23F1.5 |  |
|  |  |  |  |  |  |  |  |  |  |  |  |  |  |  |  |  |  |  |  |  |  |  |  |  |  |  |  |  |  |  |  |  |  |  |  |  |  | *bus-8* | Bacterially Un-Swollen (M. nematophilum resistant) |
|  |  |  |  |  |  |  |  |  |  |  |  |  |  |  |  |  |  |  |  |  |  |  |  |  |  |  |  |  |  |  |  |  |  |  |  |  |  | Y22D7AL.9 |  |
|  |  |  |  |  |  |  |  |  |  |  |  |  |  |  |  |  |  |  |  |  |  |  |  |  |  |  |  |  |  |  |  |  |  |  |  |  |  | *ptr-1* | PaTched Related family |
|  |  |  |  |  |  |  |  |  |  |  |  |  |  |  |  |  |  |  |  |  |  |  |  |  |  |  |  |  |  |  |  |  |  |  |  |  |  | C01H6.8 |  |
|  |  |  |  |  |  |  |  |  |  |  |  |  |  |  |  |  |  |  |  |  |  |  |  |  |  |  |  |  |  |  |  |  |  |  |  |  |  | F01G10.9 |  |
|  |  |  |  |  |  |  |  |  |  |  |  |  |  |  |  |  |  |  |  |  |  |  |  |  |  |  |  |  |  |  |  |  |  |  |  |  |  | *ptr-4* | PaTched Related family |
|  |  |  |  |  |  |  |  |  |  |  |  |  |  |  |  |  |  |  |  |  |  |  |  |  |  |  |  |  |  |  |  |  |  |  |  |  |  | *mlt-9* | MoLTing defective |
|  |  |  |  |  |  |  |  |  |  |  |  |  |  |  |  |  |  |  |  |  |  |  |  |  |  |  |  |  |  |  |  |  |  |  |  |  |  | F52E1.5 |  |
|  |  |  |  |  |  |  |  |  |  |  |  |  |  |  |  |  |  |  |  |  |  |  |  |  |  |  |  |  |  |  |  |  |  |  |  |  |  | C14H10.1 |  |
|  |  |  |  |  |  |  |  |  |  |  |  |  |  |  |  |  |  |  |  |  |  |  |  |  |  |  |  |  |  |  |  |  |  |  |  |  |  | *cuti-1* | CUTicle and epithelial Integrity |
|  |  |  |  |  |  |  |  |  |  |  |  |  |  |  |  |  |  |  |  |  |  |  |  |  |  |  |  |  |  |  |  |  |  |  |  |  |  | C15C6.1 |  |
|  |  |  |  |  |  |  |  |  |  |  |  |  |  |  |  |  |  |  |  |  |  |  |  |  |  |  |  |  |  |  |  |  |  |  |  |  |  | *acbp-3* | Acyl-Coenzyme A Binding Protein |
|  |  |  |  |  |  |  |  |  |  |  |  |  |  |  |  |  |  |  |  |  |  |  |  |  |  |  |  |  |  |  |  |  |  |  |  |  |  | F54E7.6 |  |
|  |  |  |  |  |  |  |  |  |  |  |  |  |  |  |  |  |  |  |  |  |  |  |  |  |  |  |  |  |  |  |  |  |  |  |  |  |  | *srh-22* | Serpentine Receptor, class H |
|  |  |  |  |  |  |  |  |  |  |  |  |  |  |  |  |  |  |  |  |  |  |  |  |  |  |  |  |  |  |  |  |  |  |  |  |  |  | *cex-1* | CalEXcitin |
|  |  |  |  |  |  |  |  |  |  |  |  |  |  |  |  |  |  |  |  |  |  |  |  |  |  |  |  |  |  |  |  |  |  |  |  |  |  | ZK822.5 |  |
|  |  |  |  |  |  |  |  |  |  |  |  |  |  |  |  |  |  |  |  |  |  |  |  |  |  |  |  |  |  |  |  |  |  |  |  |  |  | C50F7.9 |  |
|  |  |  |  |  |  |  |  |  |  |  |  |  |  |  |  |  |  |  |  |  |  |  |  |  |  |  |  |  |  |  |  |  |  |  |  |  |  | C06E7.88 |  |
|  |  |  |  |  |  |  |  |  |  |  |  |  |  |  |  |  |  |  |  |  |  |  |  |  |  |  |  |  |  |  |  |  |  |  |  |  |  | T05C1.3 |  |
|  |  |  |  |  |  |  |  |  |  |  |  |  |  |  |  |  |  |  |  |  |  |  |  |  |  |  |  |  |  |  |  |  |  |  |  |  |  | Y102A11A.5 |  |
|  |  |  |  |  |  |  |  |  |  |  |  |  |  |  |  |  |  |  |  |  |  |  |  |  |  |  |  |  |  |  |  |  |  |  |  |  |  | T11G6.3 |  |
|  |  |  |  |  |  |  |  |  |  |  |  |  |  |  |  |  |  |  |  |  |  |  |  |  |  |  |  |  |  |  |  |  |  |  |  |  |  | *clec-76* | C-type LECtin |
|  |  |  |  |  |  |  |  |  |  |  |  |  |  |  |  |  |  |  |  |  |  |  |  |  |  |  |  |  |  |  |  |  |  |  |  |  |  | H04M03.3 |  |
|  |  |  |  |  |  |  |  |  |  |  |  |  |  |  |  |  |  |  |  |  |  |  |  |  |  |  |  |  |  |  |  |  |  |  |  |  |  | D2023.1 |  |
|  |  |  |  |  |  |  |  |  |  |  |  |  |  |  |  |  |  |  |  |  |  |  |  |  |  |  |  |  |  |  |  |  |  |  |  |  |  | F54D11.3 |  |
|  |  |  |  |  |  |  |  |  |  |  |  |  |  |  |  |  |  |  |  |  |  |  |  |  |  |  |  |  |  |  |  |  |  |  |  |  |  | *mltn-5* | MLt-TeN (mlt-10) related |
|  |  |  |  |  |  |  |  |  |  |  |  |  |  |  |  |  |  |  |  |  |  |  |  |  |  |  |  |  |  |  |  |  |  |  |  |  |  | C14A4.13 |  |
|  |  |  |  |  |  |  |  |  |  |  |  |  |  |  |  |  |  |  |  |  |  |  |  |  |  |  |  |  |  |  |  |  |  |  |  |  |  | *try-8* | TRYpsin-like protease |
|  |  |  |  |  |  |  |  |  |  |  |  |  |  |  |  |  |  |  |  |  |  |  |  |  |  |  |  |  |  |  |  |  |  |  |  |  |  | T10G3.2 |  |
|  |  |  |  |  |  |  |  |  |  |  |  |  |  |  |  |  |  |  |  |  |  |  |  |  |  |  |  |  |  |  |  |  |  |  |  |  |  | Y37H2A.10 |  |
|  |  |  |  |  |  |  |  |  |  |  |  |  |  |  |  |  |  |  |  |  |  |  |  |  |  |  |  |  |  |  |  |  |  |  |  |  |  | F46B3.7 |  |
|  |  |  |  |  |  |  |  |  |  |  |  |  |  |  |  |  |  |  |  |  |  |  |  |  |  |  |  |  |  |  |  |  |  |  |  |  |  | *linc-94* | Long Intervening Non-Coding RNA |
|  |  |  |  |  |  |  |  |  |  |  |  |  |  |  |  |  |  |  |  |  |  |  |  |  |  |  |  |  |  |  |  |  |  |  |  |  |  | C15F1.1 |  |
|  |  |  |  |  |  |  |  |  |  |  |  |  |  |  |  |  |  |  |  |  |  |  |  |  |  |  |  |  |  |  |  |  |  |  |  |  |  | ZC449.1 |  |
|  |  |  |  |  |  |  |  |  |  |  |  |  |  |  |  |  |  |  |  |  |  |  |  |  |  |  |  |  |  |  |  |  |  |  |  |  |  | *nlp-46* | Neuropeptide-Like Protein |
|  |  |  |  |  |  |  |  |  |  |  |  |  |  |  |  |  |  |  |  |  |  |  |  |  |  |  |  |  |  |  |  |  |  |  |  |  |  | T05A7.1 |  |
|  |  |  |  |  |  |  |  |  |  |  |  |  |  |  |  |  |  |  |  |  |  |  |  |  |  |  |  |  |  |  |  |  |  |  |  |  |  | T13C5.2 |  |
|  |  |  |  |  |  |  |  |  |  |  |  |  |  |  |  |  |  |  |  |  |  |  |  |  |  |  |  |  |  |  |  |  |  |  |  |  |  | *lin-12* | abnormal cell LINeage |
|  |  |  |  |  |  |  |  |  |  |  |  |  |  |  |  |  |  |  |  |  |  |  |  |  |  |  |  |  |  |  |  |  |  |  |  |  |  | *fbxa-96* | F-box A protein |
|  |  |  |  |  |  |  |  |  |  |  |  |  |  |  |  |  |  |  |  |  |  |  |  |  |  |  |  |  |  |  |  |  |  |  |  |  |  | *kcc-1* | K+/Cl- Cotransporter |
|  |  |  |  |  |  |  |  |  |  |  |  |  |  |  |  |  |  |  |  |  |  |  |  |  |  |  |  |  |  |  |  |  |  |  |  |  |  | *pitr-4* | PIT (mammalian phosphate transporter) Related |
|  |  |  |  |  |  |  |  |  |  |  |  |  |  |  |  |  |  |  |  |  |  |  |  |  |  |  |  |  |  |  |  |  |  |  |  |  |  | Y39E4A.1 |  |
|  |  |  |  |  |  |  |  |  |  |  |  |  |  |  |  |  |  |  |  |  |  |  |  |  |  |  |  |  |  |  |  |  |  |  |  |  |  | *unc-4* | UNCoordinated |
|  |  |  |  |  |  |  |  |  |  |  |  |  |  |  |  |  |  |  |  |  |  |  |  |  |  |  |  |  |  |  |  |  |  |  |  |  |  | *ilys-4* | Invertebrate LYSozyme |

### Phenotypes enriched

|  |  |  |  |
| --- | --- | --- | --- |
| **Group name** | **Number in cluster** | **Enrichment** | **FDR corrected p** |
| alae morphology variant (RNAi) | 8 | 12.12 | 0.000399 |
| body vacuole (RNAi) | 10 | 7.84 | 0.000892 |
| male tail development variant (RNAi) | 6 | 16.47 | 0.001560 |
| intestinal vacuole (RNAi) | 10 | 7.32 | 0.001600 |
| left handed roller | 5 | 21.96 | 0.002280 |
| organism segment development variant (RNAi) | 6 | 14.64 | 0.003170 |
| tail development variant (RNAi) | 6 | 14.64 | 0.003170 |
| multiple alae (RNAi) | 6 | 14.64 | 0.003170 |
| helical cuticle | 5 | 19.97 | 0.003900 |
| cuticle morphology variant | 7 | 10.25 | 0.005800 |
| pericellular component morphology variant | 7 | 9.04 | 0.012400 |
| alae secretion variant (RNAi) | 6 | 10.98 | 0.016000 |
| struts variant | 4 | 21.96 | 0.020400 |
| roller | 5 | 13.73 | 0.024500 |
| alae morphology variant | 5 | 13.73 | 0.024500 |
| dumpy | 11 | 4.56 | 0.032500 |
| late larval lethal (RNAi) | 6 | 9.41 | 0.035900 |
| intestinal morphology variant (RNAi) | 10 | 4.83 | 0.042900 |
| alimentary system morphology variant (RNAi) | 11 | 4.39 | 0.043200 |

### Anatomy terms enriched

none found

### GO terms enriched

|  |  |  |
| --- | --- | --- |
| **GO term** | **Number of genes** | **FDR-corrected p-value** |
| structural constituent of cuticle | 20 | 2.0e-10 |
| extracellular matrix | 10 | 1.2e-05 |
| molting cycle, collagen and cuticulin-based cuticle | 17 | 2.8e-04 |
| collagen | 5 | 9.5e-03 |
| hedgehog receptor activity | 5 | 1.3e-02 |
| body morphogenesis | 21 | 2.2e-02 |
| extracellular region part | 4 | 2.4e-02 |
| positive regulation of multicellular organism growth | 14 | 4.7e-02 |
| extracellular space | 6 | 5.0e-02 |

### Expression clusters enriched

|  |  |  |  |
| --- | --- | --- | --- |
| **Group name** | **Number in cluster** | **Enrichment** | **FDR corrected p** |
| Genes significantly enriched (> 2x, FDR < 5%) in a particular cell-type versus a reference sample of all cells at the same stage. WBPaper00037950:hypodermis\_embryo\_enriched | 168 | 10.05 | 6.67e-125 |
| Genes that show selective expression in a subset of cell types vs broadly expressed in many cell types. Correspond to 20% - 57% of enriched\_genes for a given cell type. WBPaper00037950:hypodermis\_embryo\_SelectivelyEnriched | 128 | 15.71 | 9.94e-119 |
| Genes up regulated by mir-243(n4759). | 188 | 4.59 | 3.70e-79 |
| Genes significantly enriched (> 2x, FDR < 5%) in a particular cell-type versus a reference sample of all cells at both embryonic and larval stages. WBPaper00037950:hypodermis\_CoreEnriched | 78 | 19.36 | 1.24e-77 |
| Genes that show selective expression in a subset of cell types vs broadly expressed in many cell types. Correspond to 20% - 57% of enriched\_genes for a given cell type. WBPaper00037950:germline-precursors\_embryo\_SelectivelyEnriched | 115 | 9.09 | 1.87e-75 |
| Genes significantly enriched (> 2x, FDR < 5%) in a particular cell-type versus a reference sample of all cells at the same stage. WBPaper00037950:germline-precursors\_embryo\_enriched | 141 | 6.36 | 1.63e-73 |
| Genes with expression level up regulated after treatment with Methylmercury (MeHg) by RNAseq analysis. | 105 | 8.79 | 1.47e-66 |
| A cluster of genes that oscillates with the molting cycle during early larval development. | 71 | 15.21 | 2.27e-61 |
| Genes regulated by octr-1(ok371) after infected with P. aeruginosa PA14 for 4 hours at 25 centigrade. | 129 | 5.81 | 2.27e-61 |
| Genes dowm regulated in the dauer versus dauer-exit worms. | 91 | 9.13 | 2.59e-58 |
| Genes down regulated in crh-1(nn3315) comparing to in N2. | 97 | 7.88 | 2.17e-56 |
| Genes downregulated on Comamonas DA1877 relative to E. coli OP50, Young adult | 77 | 10.74 | 3.61e-54 |
| Genes with expression level down regulated in mir-35 mutants comparing with N2. | 121 | 5.24 | 5.89e-52 |
| Genes that show selective expression in a subset of cell types vs broadly expressed in many cell types. Correspond to 20% - 57% of enriched\_genes for a given cell type. WBPaper00037950:hypodermis\_larva\_SelectivelyEnriched | 93 | 6.92 | 1.05e-48 |
| A large cluster of genes up-regulated during early larval development.. | 150 | 3.59 | 2.49e-45 |
| Genes that showed decreased expression in nhr-23 RNAi experiment. | 63 | 10.56 | 4.77e-43 |
| Genes significantly enriched (> 2x, FDR < 5%) in a particular cell-type versus a reference sample of all cells at the same stage. WBPaper00037950:hypodermis\_larva\_enriched | 120 | 4.21 | 2.91e-41 |
| Expression Pattern Group G, enriched for genes involved in locomotion. | 130 | 3.82 | 1.04e-40 |
| Genes with increased expression after 24 hours of infection by P.lumniescens Fold changes shown are pathogen vs OP50. WBPaper00038438:P.lumniescens\_24hr\_upregulated\_TilingArray | 203 | 2.36 | 2.86e-37 |
| Class E gene expression showed no change in lin-14(lf) in L1, up regulation in lin-4(lf) in L2. | 64 | 8.37 | 3.39e-37 |
| Genes down regulated in the absence of TDP-1, when the threshold was set at a fold change (FC) of 1.5. | 69 | 7.08 | 1.48e-35 |
| Germline-enriched and sex-biased expression profile cluster B. | 87 | 5.08 | 2.20e-34 |
| Genes downregulated by fer-1 mutants hc1 and hc24, with > 4 fold change in expression level. | 51 | 10.23 | 1.48e-33 |
| Genes with increased expression after 24 hours of infection by S.marcescens Fold changes shown are pathogen vs OP50. WBPaper00038438:S.marcescens\_24hr\_upregulated\_RNAseq | 148 | 2.86 | 1.00e-32 |
| Genes down regulated in aak-2 over expression line uthIs202 comparing to in N2. | 57 | 7.39 | 8.35e-30 |
| Genes with increased expression after 24 hours of infection by P.lumniescens Fold changes shown are pathogen vs OP50. WBPaper00038438:P.lumniescens\_24hr\_upregulated\_RNAseq | 163 | 2.37 | 3.53e-27 |
| Genes differentially expressed in control vs after UVC exposure and EtBr treatment at the -1h timepoint (just prior to the third UVC dose (48h)). | 217 | 1.91 | 1.21e-26 |
| Genes that showed lower expression in N2 than in DR1350. | 95 | 3.56 | 1.53e-25 |
| Genes down regulated in tax-6(ok2065) comparing to in N2. | 81 | 3.71 | 2.43e-22 |
| Genes upregulated in sma-2 L4 (3 arrays) or sma-4 L4 (1 array) vs. N2 L4. | 59 | 5.00 | 7.68e-22 |
| Genes down regulated after fed by 25 mg/ml pRJ-Fr.5 for 24 h beginning at the L4 stage. | 47 | 6.51 | 9.57e-22 |
| Genes down regulated in the absence of TDP-1, when the threshold was set at a fold change (FC) of 1.2. | 129 | 2.38 | 1.05e-19 |
| Genes with expression altered >= 3-fold in osm-8(n1518) mutants. | 77 | 3.26 | 1.28e-17 |
| TGF- Dauer pathway adult transcriptional targets. Results obtained by comparing the microarray results of the dauer-constitutive mutants daf-7(e1372), daf-7(m62), and daf-1(m40) with dauer-defective mutants daf-3(mgDf90), daf-5(e1386), and daf-7(e1372);daf-3(mgDf90) double mutants at the permissive temperature, 20C, on the first day of adulthood. WBPaper00031040:TGF-beta\_adult\_upregulated | 115 | 2.32 | 3.37e-16 |
| Genes that are up or down regulated by more than 2.1 fold with the t-test p-value less than 0.01 are included in this cluster. | 82 | 2.93 | 3.91e-16 |
| Genes down-regulated after 200 um Quercetin treatment. Fold change < 0.8. | 83 | 2.80 | 3.41e-15 |
| Genes that showed decreased expression after exposure to 7.5uM CH3HgCl for 24 hours. | 77 | 2.85 | 2.43e-14 |
| Genes with expression altered >= 3-fold in dpy-9(e12) mutants. | 127 | 1.98 | 8.53e-13 |
| Developmentally modulated gene cluster. cgc4386\_cluster\_4\_6 | 20 | 10.10 | 5.15e-12 |
| Genes down-regulated after 100 um Quercetin treatment. Fold change < 0.8. | 73 | 2.64 | 9.33e-12 |
| Genes differentially expressed under EtBr treatment and UVC exposure vs under UVC exposure but without EtBr treatment at the -1h timepoint (just prior to the third UVC dose (48h)). | 157 | 1.73 | 1.05e-11 |
| Genes showing < 0.5-fold down-regulated expression (p < 0.001) both in aex-3::His-SUMO-1 or myo-4::His-SUMO-1 C. elegans. | 37 | 4.28 | 7.47e-11 |
| Genes differentially expressed in control vs after UVC exposure and EtBr treatment at the 3h timepoint (3 h after the third UVC dose (51h), which is also 3 h after being placed on food). | 150 | 1.72 | 1.27e-10 |
| Genes with decreased expression after 12 hours of infection by D.coniospora Fold changes shown are pathogen vs OP50. | 105 | 2.02 | 2.57e-10 |
| Developmentally modulated gene cluster. cgc4386\_cluster\_5\_6 | 18 | 9.41 | 3.72e-10 |
| Genome-wide analysis of developmental and sex-regulated gene expression profile. cgc4489\_group\_8 | 44 | 3.41 | 8.22e-10 |
| Genes that are upregulated by 0.2M ethanol treatment. | 65 | 2.55 | 1.55e-09 |
| Genes with expression altered >= 3-fold in dpy-10(e128) mutants. | 132 | 1.74 | 3.77e-09 |
| Genes that change expression level at L3 larva stage after ash-2 RNAi. | 24 | 5.70 | 4.23e-09 |
| Genes up regulated in alg-1(gk214) comparing to in N2. | 56 | 2.53 | 7.94e-08 |
| WT-Pico Pan-neural Depleted Genes, with genes found multiple times in a single dataset removed (without dups). | 64 | 2.24 | 4.45e-07 |
| Genes that showed lower expression in RIL-17 than in RIL-14. | 17 | 5.97 | 2.00e-06 |
| Genes predicted to be downregulated more than 2.0 fold in (AFD+AWB) datasets as compared to unsorted whole embryonic cells dataset. | 36 | 3.01 | 2.27e-06 |
| Genes differentially expressed in control vs under EtBr treatment without UVC exposure, at the -1h timepoint. | 148 | 1.52 | 2.46e-06 |
| Genes that showed decreased expression after exposure to 2.0uM CH3HgCl for 24 hours. | 19 | 4.88 | 6.69e-06 |
| Developmentally modulated gene cluster. cgc4386\_cluster\_2\_5 | 12 | 6.94 | 6.01e-05 |
| Genes with increased expression after 24 hours of infection by E.faecalis Fold changes shown are pathogen vs OP50. WBPaper00038438:E.faecalis\_24hr\_upregulated\_RNAseq | 111 | 1.57 | 8.52e-05 |
| Developmentally modulated gene cluster. cgc4386\_cluster\_2\_4 | 15 | 4.77 | 2.35e-04 |
| Genes with increased expression after 24 hours of infection by S.marcescens Fold changes shown are pathogen vs OP50. WBPaper00038438:S.marcescens\_24hr\_upregulated\_TilingArray | 97 | 1.61 | 2.41e-04 |
| Genome-wide analysis of developmental and sex-regulated gene expression profile. cgc4489\_group\_23 | 20 | 3.50 | 4.86e-04 |
| Genes upregulated by > 2-fold in CY262(sqt-1(sc13) age-1(mg44); bvIs1) adults, which intestinally express age-1, relative to wildtype. | 40 | 2.22 | 6.96e-04 |
| Genes upregulated by > 2-fold in CY251(sqt-1(sc13) age-1(mg44); bvIs2) adults, which neuronally express age-1, relative to wildtype. | 40 | 2.22 | 6.96e-04 |
| Genes upregulated by > 2-fold in SP75 (sqt-1(sc13) age-1(mg44)/mnC1) adults relative to wildtype. | 40 | 2.22 | 6.96e-04 |
| Genes Showing Significant Expression Changes (P <= 0.05 After FDR) in Heat-Shocked (30 centigrade for 1.5 h; Two Arrays), Compared to Sham Controls (26 centigrade for 2.5 h; Five Arrays). WBPaper00034636:heat\_shock\_upregulated | 7 | 10.60 | 1.06e-03 |
| Genes up-regulated more than 2-fold in let-7(n2853) compared to wild-type. | 8 | 8.37 | 1.37e-03 |
| Genes with expression level regulated by genotype (N2 vs CB4856) and age at L3 larva and Late reproduction stage (96 hours at 24 centigrade). | 120 | 1.44 | 1.53e-03 |
| Total muscle depleted genes (complete list of non-overlapping genes from the 0hr and 24hr muscle depleted datasets). | 89 | 1.57 | 1.75e-03 |
| Genes depleted in muscle cells (0hr muscle dataset). Dissociated myo-3::GFP embryos were cultured for 0 hours before FACS sorting. | 67 | 1.72 | 1.85e-03 |
| Germline-enriched and sex-biased expression profile cluster A. | 33 | 2.33 | 1.87e-03 |
| Genes down-regulated after 50 um Quercetin treatment. Fold change < 0.8. | 14 | 4.24 | 1.88e-03 |
| Genes differentially expressed under EtBr treatment and UVC exposure vs under UVC exposure but without EtBr treatment at the -3h timepoint (3 h after the third UVC dose (51h), which is also 3 h after being placed on food). | 85 | 1.58 | 2.33e-03 |
| Genes with decreased expression level after feeding with Comamonas DA1877. | 8 | 7.64 | 2.55e-03 |
| Genome-wide analysis of developmental and sex-regulated gene expression profile. cgc4489\_group\_6 | 20 | 3.01 | 3.82e-03 |
| male sex-enriched | 23 | 2.72 | 4.32e-03 |
| Candidate daf-19 down regulated genes with a statistically significant signal variation of 1.5-fold or greater. These were identified using a class comparisons tool from BRB Array Tools. | 29 | 2.35 | 5.32e-03 |
| Genes that showed expression levels higher than the corresponding reference sample (embryonic 0hr reference). WBPaper00037950:germline-precursors\_expressed | 156 | 1.27 | 2.91e-02 |
| Genes upregulated by alg-1(-). | 54 | 1.67 | 2.96e-02 |
| Genes with expression level regulated by genotype (N2 vs CB4856) at L3 larva and Late reproduction stage (96 hours at 24 centigrade). | 88 | 1.43 | 4.30e-02 |
| Genes with no change in hcf-1(-), no change in sir-2.1(O/E) and upregulated in daf-2(-). | 38 | 1.84 | 4.55e-02 |
| Genes with increased expression in sam-10(js94) mutants, revealed by microarray analysis. Geometric mean=1.5 is used as a cutoff. | 7 | 5.69 | 4.58e-02 |
| Gene significantly down-regulated by treatment with 2.0mM of HuminFeed Hydroquinone until young adult stage (3 days), with a minimum fold change in gene expression of 0.8. | 30 | 2.01 | 4.87e-02 |

### Motifs enriched

|  |  |  |  |  |  |
| --- | --- | --- | --- | --- | --- |
| **Motif** | **Logo** | **Possible orthologs** | **Number of motifs in cluster** | **Enrichment** | **FDR corrected p** |
| So\_SOLEXA\_FBgn0003460 |  | elt-3 (0.75) ceh-32 elt-6 elt-7 egl-27 ceh-34 elt-1 | 224 | 1.77 | 1.9e-21 |
| GATA1\_si |  | ztf-29 elt-1 | 218 | 1.76 | 3.1e-20 |
| GATA3\_1 |  | elt-1 end-3 | 209 | 1.81 | 3.8e-20 |
| V$GATA1\_04 |  | elt-1 | 206 | 1.80 | 1.4e-19 |
| Gata5\_3768 |  | elt-1 | 207 | 1.80 | 1.7e-19 |
| So\_Cell\_FBgn0003460 |  | ceh-32 ztf-28 skn-1 | 213 | 1.76 | 2.8e-19 |
| GRHL1\_2 |  | grh-1 | 132 | 2.35 | 1.1e-18 |
| pTH9880 |  | end-1 | 184 | 1.89 | 2.4e-18 |
| pTH1049 |  | elt-1 | 199 | 1.80 | 3.4e-18 |
| Gata3\_1024 |  | elt-1 | 195 | 1.75 | 2.0e-16 |
| Mf28 |  | elt-1 | 198 | 1.73 | 4.1e-16 |
| I$MTTFA\_01 |  | hmg-5 nsy-7 | 211 | 1.65 | 1.2e-15 |
| T-47D\_GATA3\_HudsonAlpha |  | elt-1 | 233 | 1.55 | 3.4e-15 |
| PBDE\_GATA1\_UCD |  | alr-1 elt-1 | 189 | 1.71 | 2.1e-14 |
| GATA5\_f1 |  | nhr-100 nhr-10 elt-1 nhr-7 | 245 | 1.49 | 3.5e-14 |
| MA0543.1 |  | eor-1 daf-8 | 258 | 1.42 | 4.3e-13 |
| pTH6556 |  | odd-1 lim-6 | 207 | 1.58 | 6.8e-13 |
| pTH9072 |  | klf-1 klf-2 sptf-3 | 191 | 1.64 | 9.9e-13 |
| Hlf\_1 |  | ces-2 atf-2 F23F12.9 Y51H4A.4 C48E7.11 C01B12.2 | 168 | 1.74 | 1.1e-12 |
| MA0508.1 |  | blmp-1 | 264 | 1.38 | 3.0e-12 |
| V$GATA1\_02 |  | elt-1 | 153 | 1.80 | 3.8e-12 |
| V$GATA6\_01 |  | elt-1 | 188 | 1.62 | 6.3e-12 |
| pTH10638 |  | dmd-3 C34D1.1 | 230 | 1.47 | 7.5e-12 |
| Elf3 |  | C24A1.2 | 241 | 1.44 | 8.3e-12 |
| NR4A1\_f1 |  | nhr-6 (0.54) nhr-2 nhr-68 nhr-10 nhr-213 nhr-71 Y67D8A.3 | 213 | 1.52 | 8.5e-12 |
| pTH3464 |  | nhr-69 (0.67) nhr-19 (0.55) nhr-2 nhr-10 nhr-213 | 80 | 2.55 | 9.6e-12 |
| Elf3\_3876 |  | C24A1.2 | 245 | 1.42 | 1.2e-11 |
| pTH2193 |  | nhr-2 nhr-15 nhr-213 nhr-239 | 100 | 2.20 | 1.8e-11 |
| CG5669\_SOLEXA\_5\_FBgn0039169 |  | klf-1 klf-2 | 193 | 1.58 | 2.3e-11 |
| NR1D1\_f1 |  | nhr-213 nhr-118 | 190 | 1.59 | 3.1e-11 |
| OTX1\_1 |  | ceh-53 ceh-45 pha-2 dve-1 alr-1 ceh-36 | 161 | 1.71 | 3.3e-11 |
| MA0079.3 |  | klf-1 klf-2 ZC328.2 | 204 | 1.53 | 3.3e-11 |
| I$FTZ\_01 |  | cog-1 (0.64) ceh-1 lim-7 ceh-43 ceh-8 lin-39 ceh-30 ceh-9 alr-1 ceh-24 ceh-31 ceh-19 | 193 | 1.57 | 4.7e-11 |
| I$E74A\_01 |  | lin-1 C24A1.2 | 241 | 1.42 | 4.9e-11 |
| Elf5 |  | C24A1.2 | 234 | 1.43 | 5.2e-11 |
| ETS2\_f1 |  | lin-1 C24A1.2 | 229 | 1.45 | 6.7e-11 |
| pTH9237 |  | mel-28 | 265 | 1.35 | 6.7e-11 |
| MA0452.2 |  | B0310.2 (0.82) ZK177.3 | 233 | 1.43 | 1.3e-10 |
| V$GATA3\_01 |  | elt-1 | 179 | 1.60 | 1.5e-10 |
| pTH2283 |  | odd-2 | 236 | 1.42 | 1.6e-10 |
| MA0167.1 |  | dsc-1 (0.72) cog-1 (0.64) egl-5 (0.62) ceh-1 ceh-53 ceh-14 lim-7 ceh-18 pal-1 ceh-43 lim-4 lin-39 ceh-45 ceh-10 pha-2 ceh-30 ceh-16 alr-1 ceh-36 ceh-2 and 9 others  [full list] | 208 | 1.50 | 1.7e-10 |
| HXA9\_f1 |  | ceh-32 lin-39 | 208 | 1.50 | 1.7e-10 |
| Msx3\_1 |  | ceh-1 ceh-14 ceh-43 lin-39 ceh-31 | 233 | 1.42 | 1.8e-10 |
| K562\_ELF1\_HudsonAlpha |  | lin-1 C24A1.2 | 222 | 1.45 | 1.9e-10 |
| MA0473.1 |  | lin-1 C24A1.2 | 243 | 1.39 | 2.2e-10 |
| pTH6445 |  | ceh-5 | 219 | 1.46 | 2.8e-10 |
| PBX1\_do |  | ceh-20 lin-39 ceh-12 | 245 | 1.39 | 2.8e-10 |
| Ara\_SOLEXA\_FBgn0015904 |  | fkh-9 irx-1 daf-16 | 219 | 1.46 | 3.0e-10 |
| MA0246.1 |  | ceh-32 dmd-5 dmd-4 | 141 | 1.77 | 3.3e-10 |
| disco-r-Cl1\_SANGER\_5\_FBgn0042650 |  | lin-31 (0.69) nhr-68 F55C5.11 | 205 | 1.49 | 4.3e-10 |
| V$AREB6\_02 |  | ztf-6 | 189 | 1.54 | 4.6e-10 |
| Tcf7l2\_3461 |  | ceh-20 pop-1 | 242 | 1.38 | 5.9e-10 |
| NR2F1\_1 |  | nhr-19 (0.55) nhr-2 nhr-62 | 187 | 1.55 | 6.7e-10 |
| NR2F6\_f1 |  | nhr-2 nhr-62 nhr-239 | 254 | 1.35 | 6.9e-10 |
| Six1\_0935 |  | ceh-32 | 132 | 1.80 | 7.4e-10 |
| V$CREB\_01 |  | crh-1 W08E12.1 | 174 | 1.59 | 7.7e-10 |
| SRP000712\_Sox2 |  | ceh-18 ceh-6 sox-4 tbp-1 | 247 | 1.37 | 9.4e-10 |
| NR2E3\_f1 |  | lin-1 nhr-100 lin-39 | 247 | 1.37 | 1.0e-09 |
| sqz\_SOLEXA\_5\_FBgn0010768 |  | mel-28 fkh-7 lin-29 | 263 | 1.33 | 1.0e-09 |
| TBX3\_f1 |  | ceh-45 tbx-39 | 220 | 1.43 | 1.4e-09 |
| ems\_FlyReg\_FBgn0000576 |  | ceh-2 skn-1 | 189 | 1.52 | 1.5e-09 |
| Cutl1\_3494 |  | ceh-44 | 168 | 1.60 | 1.5e-09 |
| PHOX2B\_2 |  | cfi-1 ceh-14 alr-1 ZC204.2 | 203 | 1.48 | 1.6e-09 |
| V$OCT1\_03 |  | ceh-18 | 191 | 1.51 | 1.9e-09 |
| Hoxc10\_2 |  | php-3 pal-1 lin-39 ceh-24 | 261 | 1.33 | 2.0e-09 |
| Phox2b\_3948 |  | ceh-1 ceh-53 ceh-18 npax-3 ceh-43 lin-39 ceh-45 alr-1 ceh-12 | 182 | 1.54 | 2.1e-09 |
| pTH5082 |  | atf-2 ceh-16 | 175 | 1.57 | 2.1e-09 |
| Mv75 |  | lin-31 (0.69) elt-1 | 141 | 1.72 | 2.1e-09 |
| ARI3A\_do |  | cfi-1 nhr-208 gei-3 lim-6 | 292 | 1.26 | 2.2e-09 |
| FOXJ3\_f2 |  | lin-31 (0.69) let-381 fkh-8 daf-16 pha-4 fkh-7 fkh-10 | 289 | 1.26 | 2.4e-09 |
| pTH9059 |  | che-1 (0.52) ztf-28 ces-1 | 55 | 2.87 | 2.5e-09 |
| Hoxc4\_3491 |  | lin-39 | 191 | 1.51 | 2.5e-09 |
| TBX20\_4 |  | mab-9 tbx-38 tbx-39 tbx-43 tbx-42 | 194 | 1.50 | 2.8e-09 |
| TBP\_f1 |  | tbp-1 | 194 | 1.50 | 2.8e-09 |
| MA0488.1 |  | crh-1 ceh-26 | 204 | 1.47 | 2.9e-09 |
| BSX\_1 |  | ceh-1 lim-7 ceh-43 lin-39 ceh-9 ceh-16 alr-1 ceh-31 | 179 | 1.55 | 3.1e-09 |
| pTH9261 |  | lin-48 (0.52) dmd-3 pax-3 | 216 | 1.43 | 3.2e-09 |
| pTH5437 |  | ceh-34 | 133 | 1.75 | 3.3e-09 |
| Hoxb4\_2627 |  | lin-39 | 191 | 1.50 | 3.4e-09 |
| Meis1\_2335 |  | ceh-32 ceh-20 ces-1 F55C5.11 | 200 | 1.47 | 3.8e-09 |
| CG31670\_SOLEXA\_5\_FBgn0031375 |  | CELE\_Y38H8A.5 | 239 | 1.37 | 4.8e-09 |
| Hoxa13\_3126 |  | pal-1 ceh-24 | 224 | 1.40 | 4.8e-09 |
| Hoxa4\_3426 |  | lin-39 | 187 | 1.51 | 4.9e-09 |
| HOXC10\_1 |  | php-3 hbl-1 lin-39 | 225 | 1.40 | 5.0e-09 |
| pTH8998 |  | mab-3 | 211 | 1.44 | 6.1e-09 |
| retn\_SANGER\_5\_FBgn0004795 |  | let-381 cfi-1 ceh-20 | 239 | 1.36 | 6.6e-09 |
| Vax1\_3499 |  | C02F12.10 | 180 | 1.53 | 7.5e-09 |
| ZN384\_f1 |  | K11D2.4 lin-29 | 277 | 1.28 | 7.7e-09 |
| nau\_da\_SANGER\_5\_FBgn0002922 |  | hlh-1 (0.63) K02D7.2 (0.53) hlh-15 ces-1 hlh-2 | 194 | 1.48 | 8.4e-09 |
| V$MEF2\_04 |  | mef-2 | 216 | 1.41 | 1.1e-08 |
| rn\_SOLEXA\_5\_FBgn0259172 |  | lin-29 | 288 | 1.25 | 1.1e-08 |
| pTH5781 |  | ceh-32 | 197 | 1.47 | 1.1e-08 |
| HOXA10\_1 |  | php-3 pal-1 lin-39 ceh-24 ceh-13 D1005.3 T27F2.4 | 208 | 1.43 | 1.2e-08 |
| Cdx1\_2245 |  | ceh-13 | 208 | 1.43 | 1.2e-08 |
| pTH9907 |  | nhr-34 | 204 | 1.45 | 1.2e-08 |
| pTH10630 |  | lsy-27 | 190 | 1.49 | 1.3e-08 |
| Elf4 |  | C24A1.2 | 193 | 1.48 | 1.3e-08 |
| pTH3510 |  | F13H6.1 (0.54) nhr-177 nhr-2 nhr-86 | 162 | 1.58 | 1.4e-08 |
| pTH9214 |  | cfi-1 | 235 | 1.36 | 1.5e-08 |
| Six6\_2267 |  | ceh-32 ceh-34 | 117 | 1.81 | 1.7e-08 |
| Sry\_2833 |  | pop-1 gei-3 sox-4 C05C9.3 | 204 | 1.44 | 1.7e-08 |
| pTH9384 |  | cfi-1 | 251 | 1.33 | 1.8e-08 |
| pTH3998 |  | tbx-39 | 193 | 1.47 | 2.1e-08 |
| Antp\_Cell\_FBgn0000095 |  | php-3 unc-86 cfi-1 lim-7 lin-39 | 195 | 1.46 | 2.1e-08 |
| Mw160 |  | nhr-68 | 227 | 1.38 | 2.2e-08 |
| MA0235.1 |  | dsc-1 (0.72) ceh-48 | 237 | 1.35 | 2.3e-08 |
| Nkx1-1\_3856 |  | ceh-30 | 163 | 1.56 | 2.6e-08 |
| Sox4 |  | nhr-100 pop-1 sox-4 | 249 | 1.32 | 2.7e-08 |
| pTH9296 |  | ztf-6 C34D1.1 gei-11 | 240 | 1.34 | 2.7e-08 |
| pTH5690 |  | ceh-32 | 159 | 1.58 | 2.9e-08 |
| Mcm1 |  | unc-120 (0.59) | 185 | 1.49 | 3.0e-08 |
| pTH10647 |  | nhr-232 | 190 | 1.47 | 3.7e-08 |
| pTH9215 |  | C34D1.1 | 220 | 1.39 | 3.7e-08 |
| Hoxa5\_3415 |  | lin-39 | 176 | 1.51 | 4.0e-08 |
| pTH9924 |  | nhr-46 | 233 | 1.36 | 4.2e-08 |
| pTH9125 |  | egl-13 K11D2.4 sox-4 | 240 | 1.34 | 4.3e-08 |
| V$NGFIC\_01 |  | klf-1 klf-2 ZC328.2 | 179 | 1.50 | 5.0e-08 |
| pTH9163 |  | nhr-3 | 211 | 1.41 | 5.1e-08 |
| MA0249.1 |  | hlh-8 (0.69) lin-31 (0.69) hlh-32 hlh-15 ngn-1 | 212 | 1.40 | 6.0e-08 |
| pTH10041 |  | ztf-29 | 166 | 1.54 | 6.2e-08 |
| ELF3\_f1 |  | unc-120 (0.59) K02D7.2 (0.53) C24A1.2 | 211 | 1.40 | 6.2e-08 |
| K562\_SRF\_HudsonAlpha |  | unc-120 (0.59) hlh-10 | 157 | 1.57 | 6.3e-08 |
| Elf2 |  | lin-1 F19F10.1 C24A1.2 | 189 | 1.46 | 6.3e-08 |
| V$TCF11\_01 |  | skn-1 | 197 | 1.44 | 6.3e-08 |
| pTH5119 |  | cfi-1 | 158 | 1.57 | 6.4e-08 |
| MA0262.1 |  | hsf-1 mab-3 | 229 | 1.36 | 6.6e-08 |
| pTH5561 |  | nhr-239 | 189 | 1.46 | 7.5e-08 |
| CDC5L\_si |  | D1081.8 hlh-32 irx-1 | 171 | 1.51 | 7.7e-08 |
| pTH10714 |  | nhr-216 nhr-84 nhr-142 | 200 | 1.43 | 7.8e-08 |
| Nkx1-2\_3214 |  | ceh-30 | 163 | 1.54 | 9.1e-08 |
| pTH9242 |  | mel-28 | 258 | 1.29 | 9.5e-08 |
| V$FOXO1\_02 |  | lin-31 (0.69) let-381 fkh-8 daf-16 fkh-7 fkh-10 | 252 | 1.30 | 9.6e-08 |
| V$CETS1P54\_02 |  | C52B9.2 | 217 | 1.38 | 9.7e-08 |
| Vax2\_3500 |  | C02F12.10 | 174 | 1.50 | 1.0e-07 |
| Pou2f2\_3748 |  | lim-7 ceh-18 alr-1 | 194 | 1.44 | 1.2e-07 |
| pTH9198 |  | dmd-3 | 186 | 1.46 | 1.2e-07 |
| CG7386\_F10-12\_SANGER\_5\_FBgn0035691 |  | F56D1.1 gei-3 sox-4 | 205 | 1.41 | 1.2e-07 |
| pTH2684 |  | fos-1 | 183 | 1.46 | 1.4e-07 |
| dys\_tgo\_SANGER\_5\_FBgn0015014 |  | hlh-30 pax-1 aha-1 | 180 | 1.47 | 1.5e-07 |
| I$UBX\_01 |  | lin-39 | 158 | 1.55 | 1.5e-07 |
| Caup\_SOLEXA\_FBgn0015919 |  | irx-1 | 158 | 1.55 | 1.5e-07 |
| pTH9096 |  | T07C12.11 | 43 | 2.98 | 1.6e-07 |
| Barx1\_2877 |  | ceh-43 | 170 | 1.50 | 1.7e-07 |
| pTH8991 |  | cey-3 | 175 | 1.49 | 1.8e-07 |
| Abd-A\_FlyReg\_FBgn0000014 |  | ceh-1 lin-39 ceh-45 ceh-10 alr-1 eyg-1 | 199 | 1.42 | 1.9e-07 |
| Tcf3\_3787 |  | pop-1 | 209 | 1.39 | 2.0e-07 |
| MA0254.1 |  | unc-86 ceh-18 | 192 | 1.43 | 2.0e-07 |
| pTH9246 |  | lin-31 (0.69) let-381 fkh-10 C34D1.1 | 213 | 1.38 | 2.2e-07 |
| V$FAC1\_01 |  | gei-8 | 259 | 1.28 | 2.2e-07 |
| pTH5078 |  | ces-2 | 108 | 1.79 | 2.3e-07 |
| Hmx1\_3423 |  | ceh-9 | 195 | 1.42 | 2.4e-07 |
| SOX2\_6 |  | grh-1 dmd-4 sox-4 | 165 | 1.51 | 2.5e-07 |
| Nkx6-1\_2825 |  | cog-1 (0.64) | 193 | 1.43 | 2.6e-07 |
| ZNF75A\_1 |  | ztf-3 lag-1 F26F4.8 | 229 | 1.34 | 2.8e-07 |
| pTH3041 |  | atf-2 | 155 | 1.54 | 2.9e-07 |
| MA0161.1 |  | nfi-1 F49E12.6 | 115 | 1.73 | 2.9e-07 |
| CG4854\_SANGER\_10\_FBgn0038766 |  | K11D2.4 | 222 | 1.35 | 3.2e-07 |
| pTH9135 |  | pop-1 | 243 | 1.31 | 3.3e-07 |
| pTH9915 |  | zip-3 (0.53) crh-1 | 184 | 1.45 | 3.3e-07 |
| pTH9149 |  | ztf-30 (0.72) | 123 | 1.68 | 3.3e-07 |
| TBX2\_f1 |  | ztf-6 tbx-39 | 193 | 1.42 | 3.4e-07 |
| pTH5928 |  | ceh-34 | 146 | 1.57 | 3.4e-07 |
| pTH6016 |  | ceh-18 pal-1 ceh-43 lin-39 alr-1 ceh-12 | 207 | 1.38 | 3.9e-07 |
| pTH8399 |  | lin-54 | 236 | 1.32 | 4.3e-07 |
| Barx2\_3447 |  | ceh-43 | 178 | 1.46 | 4.4e-07 |
| Hoxd10\_2368 |  | php-3 lin-39 | 216 | 1.36 | 4.6e-07 |
| Pbx1\_3203 |  | ceh-20 | 205 | 1.39 | 4.6e-07 |
| pTH10633 |  | R07H5.10 C48E7.11 | 252 | 1.29 | 4.9e-07 |
| pTH10722 |  | eor-1 ref-2 egrh-3 | 169 | 1.48 | 5.7e-07 |
| pTH5462 |  | jun-1 fos-1 sknr-1 crh-1 F45H11.6 | 162 | 1.50 | 5.9e-07 |
| pTH3751 |  | tbx-39 | 128 | 1.64 | 5.9e-07 |
| Hoxb3\_1720 |  | lin-39 | 174 | 1.46 | 6.0e-07 |
| pTH6562 |  | ceh-5 | 170 | 1.48 | 6.1e-07 |
| pTH9300 |  | dmd-3 C34D1.1 | 181 | 1.44 | 6.1e-07 |
| Jundm2\_0911 |  | fos-1 skn-1 | 182 | 1.44 | 7.4e-07 |
| V$IK2\_01 |  | F26F4.8 | 179 | 1.45 | 7.5e-07 |
| Tcf7\_0950 |  | pop-1 | 248 | 1.29 | 7.8e-07 |
| pTH9044 |  | bed-3 nhr-177 mbr-1 F26F4.8 | 161 | 1.50 | 8.5e-07 |
| V$VMYB\_01 |  | B0310.2 (0.82) lin-48 (0.52) D1081.8 | 172 | 1.46 | 8.6e-07 |
| HAND1\_si |  | hlh-8 (0.69) daf-8 sma-4 | 188 | 1.41 | 1.1e-06 |
| pTH9097 |  | Y116A8C.22 | 284 | 1.22 | 1.1e-06 |
| HXA7\_f1 |  | lin-39 elt-1 | 186 | 1.42 | 1.1e-06 |
| pTH9080 |  | mnm-2 | 176 | 1.45 | 1.2e-06 |
| pTH5118 |  | cfi-1 | 205 | 1.37 | 1.3e-06 |
| pTH10816 |  | dmd-6 | 244 | 1.29 | 1.3e-06 |
| pTH6449 |  | ceh-43 | 167 | 1.47 | 1.3e-06 |
| MA0102.3 |  | C48E7.11 | 223 | 1.33 | 1.3e-06 |
| MA0253.1 |  | dsc-1 (0.72) ceh-24 ceh-22 | 222 | 1.33 | 1.3e-06 |
| Cdx2\_4272 |  | ceh-13 | 204 | 1.37 | 1.4e-06 |
| Smad3\_3805 |  | daf-8 | 179 | 1.43 | 1.5e-06 |
| pTH6447 |  | ceh-19 | 182 | 1.42 | 1.5e-06 |
| pTH10028 |  | nhr-204 | 185 | 1.42 | 1.5e-06 |
| pTH9216 |  | ceh-18 | 173 | 1.45 | 1.5e-06 |
| pTH9708 |  | ceh-34 | 275 | 1.23 | 1.6e-06 |
| pTH6569 |  | ceh-43 | 164 | 1.48 | 1.6e-06 |
| FOXO6\_3 |  | ZC328.2 daf-16 | 184 | 1.41 | 1.9e-06 |
| pTH9951 |  | mex-6 pal-1 | 154 | 1.50 | 1.9e-06 |
| FLI1\_f1 |  | lin-1 | 176 | 1.44 | 2.0e-06 |
| pTH9969 |  | pag-3 | 218 | 1.33 | 2.1e-06 |
| pTH9314 |  | fos-1 atf-6 atf-7 | 174 | 1.44 | 2.2e-06 |
| MA0042.1 |  | lin-31 (0.69) let-381 | 245 | 1.28 | 2.3e-06 |
| pTH9182 |  | tbx-39 | 119 | 1.64 | 2.5e-06 |
| Sox17\_2837 |  | sox-4 | 237 | 1.29 | 2.6e-06 |
| pTH10718 |  | egl-43 | 188 | 1.40 | 2.7e-06 |
| pTH5083 |  | atf-5 (0.57) fos-1 crh-1 | 170 | 1.45 | 2.7e-06 |
| Six3\_1732 |  | ceh-34 | 141 | 1.54 | 2.7e-06 |
| pTH6071 |  | C46E10.8 C33G8.2 | 174 | 1.43 | 2.7e-06 |
| CG14962\_SANGER\_5\_FBgn0035407 |  | ces-1 ceh-24 C34H4.5 T22H9.4 | 210 | 1.35 | 2.8e-06 |
| I$CROC\_01 |  | let-381 mel-28 mef-2 Y116A8C.22 Y61A9LA.9 | 251 | 1.27 | 3.1e-06 |
| Irx3\_1 |  | irx-1 | 221 | 1.32 | 3.9e-06 |
| Pknox2\_3077 |  | ceh-32 | 172 | 1.43 | 3.9e-06 |
| Hoxa3\_2783 |  | lin-39 | 166 | 1.45 | 3.9e-06 |
| Gsh2\_3990 |  | ceh-31 | 165 | 1.45 | 4.1e-06 |
| EMX2\_2 |  | ceh-16 ceh-2 | 211 | 1.34 | 4.3e-06 |
| pTH5778 |  | egl-5 (0.62) | 185 | 1.40 | 4.4e-06 |
| Sox15\_3457 |  | sox-4 | 185 | 1.40 | 4.5e-06 |
| pTH10823 |  | B0310.2 (0.82) | 175 | 1.42 | 4.6e-06 |
| Evx1\_3952 |  | ceh-53 | 158 | 1.47 | 5.0e-06 |
| pTH9381 |  | ceh-18 | 171 | 1.43 | 5.2e-06 |
| MA0502.1 |  | lin-31 (0.69) ceh-20 cey-3 dro-1 | 180 | 1.41 | 5.3e-06 |
| pTH5250 |  | C48E7.11 | 133 | 1.56 | 5.3e-06 |
| Etv3 |  | lin-1 | 197 | 1.36 | 5.5e-06 |
| pTH5099 |  | hlh-1 (0.63) hlh-11 lin-32 hlh-15 hlh-14 | 190 | 1.38 | 5.5e-06 |
| Lhx4\_1719 |  | lim-7 ceh-16 ceh-24 lim-6 | 186 | 1.39 | 5.6e-06 |
| Lhx1\_2240 |  | lim-7 | 185 | 1.39 | 5.9e-06 |
| CG8765\_SANGER\_5\_FBgn0036900 |  | H20J04.3 | 223 | 1.31 | 6.0e-06 |
| Atf1\_3026 |  | crh-1 | 288 | 1.20 | 6.3e-06 |
| V$OCT1\_06 |  | ztf-9 ceh-18 | 241 | 1.27 | 6.5e-06 |
| Hr46\_FlyReg\_FBgn0000448 |  | lin-31 (0.69) nhr-213 | 168 | 1.43 | 6.6e-06 |
| pTH1014 |  | atf-5 (0.57) | 26 | 3.69 | 6.7e-06 |
| pTH9049 |  | ztf-2 ztf-6 | 209 | 1.34 | 6.7e-06 |
| pTH9222 |  | mel-28 | 243 | 1.27 | 6.8e-06 |
| pTH9353 |  | ceh-51 | 143 | 1.51 | 7.2e-06 |
| pTH9177 |  | hsf-1 F10B5.3 Y53C10A.3 | 185 | 1.39 | 7.7e-06 |
| pTH10795 |  | ztf-14 lin-1 ZC328.2 pax-3 ref-2 | 128 | 1.57 | 8.0e-06 |
| Hoxb5\_3122 |  | lin-39 | 163 | 1.44 | 8.1e-06 |
| Hoxa11\_2218 |  | php-3 | 161 | 1.45 | 8.2e-06 |
| pTH9365 |  | ceh-18 lin-39 ceh-6 | 143 | 1.51 | 8.4e-06 |
| pTH1001 |  | dnj-17 | 151 | 1.48 | 8.4e-06 |
| Meox1\_2310 |  | ceh-31 | 175 | 1.41 | 8.9e-06 |
| pTH3819 |  | ceh-18 | 143 | 1.50 | 9.0e-06 |
| Hoxc8\_3429 |  | lin-39 | 151 | 1.48 | 9.2e-06 |
| MA0095.2 |  | lsy-2 | 214 | 1.32 | 9.7e-06 |
| pTH9254 |  | mel-28 | 246 | 1.26 | 1.0e-05 |
| pTH9244 |  | tbx-39 | 178 | 1.40 | 1.1e-05 |
| Hoxd11\_3873 |  | php-3 ceh-13 | 173 | 1.41 | 1.1e-05 |
| Nkx6-3\_3446 |  | cog-1 (0.64) | 184 | 1.38 | 1.2e-05 |
| pTH10805 |  | ztf-16 | 167 | 1.42 | 1.2e-05 |
| PLAG1\_si |  | Y53H1A.2 plp-2 | 132 | 1.54 | 1.2e-05 |
| Six4\_2860 |  | ceh-32 | 117 | 1.60 | 1.2e-05 |
| V$BRN2\_01 |  | ceh-18 | 254 | 1.24 | 1.2e-05 |
| Emx2\_3420 |  | ceh-2 | 153 | 1.46 | 1.3e-05 |
| V$S8\_01 |  | ceh-45 | 196 | 1.35 | 1.3e-05 |
| Hoxa2\_3079 |  | lin-39 | 168 | 1.42 | 1.3e-05 |
| pTH2820 |  | ZC328.2 | 268 | 1.22 | 1.4e-05 |
| En1\_3123 |  | ceh-53 ceh-16 | 182 | 1.38 | 1.5e-05 |
| Hoxa7\_3750 |  | lin-39 | 148 | 1.47 | 1.5e-05 |
| pTH5714 |  | nhr-239 | 158 | 1.44 | 1.8e-05 |
| pTH9082 |  | mab-23 | 265 | 1.22 | 1.9e-05 |
| Bsx\_3483 |  | ceh-31 | 159 | 1.43 | 2.0e-05 |
| pTH5919 |  | irx-1 | 143 | 1.48 | 2.1e-05 |
| BARHL2\_1 |  | ceh-31 | 178 | 1.38 | 2.1e-05 |
| pTH9925 |  | nhr-100 ztf-11 | 209 | 1.32 | 2.2e-05 |
| Hoxa6\_1040 |  | lin-39 | 178 | 1.38 | 2.4e-05 |
| pTH10013 |  | nhr-168 | 203 | 1.33 | 2.4e-05 |
| Dlx2\_2273 |  | ceh-43 | 175 | 1.39 | 2.6e-05 |
| V$DELTAEF1\_01 |  | ztf-6 hlh-2 | 163 | 1.42 | 2.7e-05 |
| pTH8671 |  | attf-1 C01B12.2 | 95 | 1.70 | 2.8e-05 |
| pTH9137 |  | nhr-65 | 253 | 1.24 | 3.0e-05 |
| Zbtb12\_2932 |  | ceh-90 lsy-27 | 136 | 1.50 | 3.0e-05 |
| Tbp\_pr781 |  | tbp-1 | 115 | 1.58 | 3.2e-05 |
| Dlx1\_1741 |  | ceh-43 | 176 | 1.38 | 3.3e-05 |
| Hoxd13\_2356 |  | pal-1 | 216 | 1.30 | 3.6e-05 |
| Hlxb9\_3422 |  | ceh-12 | 160 | 1.42 | 3.6e-05 |
| Hoxa10\_2318 |  | ceh-24 | 194 | 1.34 | 3.7e-05 |
| Irx5\_2385 |  | irx-1 | 162 | 1.41 | 3.7e-05 |
| Irx3\_0920 |  | irx-1 | 145 | 1.46 | 3.8e-05 |
| pTH6436 |  | ceh-53 | 155 | 1.43 | 3.8e-05 |
| HeLa-S3\_ZNF274\_UCD |  | C28G1.4 (-0.51) | 208 | 1.31 | 3.9e-05 |
| Dlx3\_1030 |  | ceh-43 | 173 | 1.38 | 4.1e-05 |
| YY1\_1 |  | lsy-2 | 177 | 1.37 | 4.2e-05 |
| Sox1\_2631 |  | sox-4 | 216 | 1.29 | 4.3e-05 |
| V$YY1\_01 |  | lsy-2 | 206 | 1.31 | 4.7e-05 |
| pTH6482 |  | ceh-19 | 158 | 1.42 | 4.8e-05 |
| ECC-1\_ERALPHA\_HudsonAlpha |  | nhr-43 (0.89) nhr-6 (0.54) nhr-71 | 259 | 1.22 | 4.9e-05 |
| pTH10788 |  | tbx-33 | 215 | 1.29 | 5.0e-05 |
| MA0451.1 |  | php-3 nhr-2 | 162 | 1.40 | 5.4e-05 |
| Lbx2\_3869 |  | mls-2 | 146 | 1.45 | 5.8e-05 |
| V$PAX6\_01 |  | pax-2 pax-3 | 202 | 1.31 | 6.1e-05 |
| Hoxd1\_3448 |  | ceh-12 | 153 | 1.42 | 6.1e-05 |
| pTH6641 |  | lin-31 (0.69) | 234 | 1.26 | 6.6e-05 |
| MA0118.1 |  | ref-2 | 137 | 1.47 | 6.7e-05 |
| pTH3477 |  | daf-16 | 230 | 1.26 | 7.7e-05 |
| pTH6423 |  | pha-2 | 165 | 1.39 | 7.8e-05 |
| pTH10777 |  | dmd-3 | 190 | 1.33 | 7.9e-05 |
| pTH10629 |  | daf-12 (0.53) ztf-3 hif-1 ceh-9 | 191 | 1.33 | 8.0e-05 |
| pTH9709 |  | die-1 | 207 | 1.30 | 8.4e-05 |
| Hoxa7\_2668 |  | lin-39 | 163 | 1.39 | 8.7e-05 |
| Pou2f3\_3986 |  | ceh-18 | 193 | 1.32 | 9.4e-05 |
| pTH3997 |  | C04F5.9 | 133 | 1.47 | 1.0e-04 |
| V$GATA1\_01 |  | elt-1 | 130 | 1.48 | 1.1e-04 |
| Mafk\_3106 |  | F45H11.6 | 223 | 1.26 | 1.2e-04 |
| pTH5257 |  | C48E7.11 | 115 | 1.53 | 1.3e-04 |
| pTH1739 |  | nhr-255 npax-1 lin-14 | 173 | 1.36 | 1.3e-04 |
| pTH9220 |  | mbr-1 | 212 | 1.28 | 1.4e-04 |
| tgo\_ss\_SANGER\_5\_FBgn0015014 |  | aha-1 | 145 | 1.43 | 1.4e-04 |
| pTH10650 |  | nhr-153 | 162 | 1.38 | 1.5e-04 |
| HepG2\_RFX5\_Stanford |  | daf-19 | 162 | 1.38 | 1.5e-04 |
| pTH10623 |  | scrt-1 | 71 | 1.81 | 1.5e-04 |
| pTH10656 |  | hlh-8 (0.69) hlh-32 hlh-15 hlh-12 ngn-1 | 187 | 1.32 | 1.5e-04 |
| Hoxb8\_3780 |  | lin-39 | 166 | 1.37 | 1.6e-04 |
| pTH9260 |  | mel-28 | 263 | 1.20 | 1.8e-04 |
| pTH9387 |  | C34D1.1 | 128 | 1.47 | 1.8e-04 |
| Vsx1\_1728 |  | alr-1 | 169 | 1.36 | 1.9e-04 |
| V$MYB\_Q6 |  | D1081.8 | 154 | 1.39 | 1.9e-04 |
| pTH9189 |  | dmd-3 ceh-18 | 209 | 1.28 | 2.0e-04 |
| pTH6591 |  | lin-31 (0.69) | 239 | 1.23 | 2.0e-04 |
| HEN1\_si |  | hlh-1 (0.63) hlh-15 | 247 | 1.22 | 2.2e-04 |
| pTH6478 |  | lim-7 | 156 | 1.38 | 2.3e-04 |
| V$FREAC7\_01 |  | lin-31 (0.69) | 229 | 1.24 | 2.3e-04 |
| pTH10808 |  | ztf-19 | 84 | 1.67 | 2.4e-04 |
| pTH10779 |  | nhr-134 nhr-182 | 189 | 1.31 | 2.5e-04 |
| pTH2846 |  | lin-31 (0.69) | 232 | 1.24 | 2.6e-04 |
| pTH8649 |  | mbr-1 | 209 | 1.27 | 2.7e-04 |
| V$CDPCR1\_01 |  | ceh-48 | 157 | 1.37 | 3.1e-04 |
| TLX1\_f1 |  | ceh-19 | 277 | 1.17 | 3.2e-04 |
| pTH9245 |  | ceh-18 | 196 | 1.29 | 3.2e-04 |
| Srf\_3509 |  | unc-120 (0.59) | 156 | 1.38 | 3.2e-04 |
| pTH9043 |  | sem-2 | 204 | 1.28 | 3.7e-04 |
| pTH9297 |  | ceh-18 | 170 | 1.34 | 3.7e-04 |
| K562\_ZBTB7A\_HudsonAlpha |  | ZC328.2 | 137 | 1.42 | 3.7e-04 |
| Pou3f1\_3819 |  | ceh-6 | 150 | 1.39 | 3.8e-04 |
| FOXD2\_1 |  | lin-31 (0.69) let-381 ceh-53 hmg-12 ceh-43 hmbx-1 lin-39 Y116A8C.22 | 234 | 1.23 | 4.2e-04 |
| V$CDPCR3\_01 |  | ceh-48 | 172 | 1.33 | 4.3e-04 |
| FLI1\_4 |  | lin-1 | 154 | 1.37 | 4.3e-04 |
| pTH7875 |  | mel-28 | 156 | 1.37 | 4.3e-04 |
| Gmeb1\_1745 |  | attf-1 | 53 | 1.96 | 4.5e-04 |
| Pou3f4\_3773 |  | ceh-6 | 155 | 1.37 | 4.8e-04 |
| RFX3\_f1 |  | daf-19 | 162 | 1.35 | 5.3e-04 |
| pTH9026 |  | attf-1 | 82 | 1.65 | 5.3e-04 |
| V$HOX13\_01 |  | lin-39 | 171 | 1.33 | 5.5e-04 |
| Pou3f3\_3235 |  | ceh-6 | 146 | 1.39 | 5.7e-04 |
| Irx2\_0900 |  | irx-1 | 142 | 1.39 | 7.5e-04 |
| pTH2936 |  | nhr-239 | 286 | 1.15 | 8.0e-04 |
| MA0163.1 |  | Y53H1A.2 D1081.8 | 141 | 1.39 | 8.0e-04 |
| pTH5812 |  | ceh-14 | 145 | 1.38 | 8.3e-04 |
| pTH9164 |  | ceh-26 | 104 | 1.50 | 9.9e-04 |
| pTH1292 |  | pzf-1 (-0.65) | 186 | 1.29 | 1.1e-03 |
| MA0535.1 |  | pax-2 daf-8 | 142 | 1.38 | 1.1e-03 |
| pTH10027 |  | M03D4.4 | 129 | 1.41 | 1.1e-03 |
| pTH9335 |  | mel-28 | 246 | 1.20 | 1.2e-03 |
| pTH3064 |  | crh-1 | 288 | 1.14 | 1.2e-03 |
| HepG2\_HSF1\_Stanford |  | Y53C10A.3 | 150 | 1.35 | 1.3e-03 |
| MCR\_f1 |  | nhr-255 | 158 | 1.33 | 1.3e-03 |
| pTH9256 |  | ceh-18 | 166 | 1.32 | 1.4e-03 |
| pTH5914 |  | attf-1 | 12 | 4.83 | 1.5e-03 |
| pTH8983 |  | tag-347 | 130 | 1.40 | 1.5e-03 |
| Spdef\_0905 |  | lin-1 | 130 | 1.40 | 1.6e-03 |
| MA0456.1 |  | ref-2 | 127 | 1.40 | 1.6e-03 |
| pTH9219 |  | xbp-1 C01B12.2 | 49 | 1.91 | 1.8e-03 |
| CENPB\_1 |  | F21D5.4 F52B11.1 | 131 | 1.39 | 1.9e-03 |
| pTH5976 |  | hlh-30 irx-1 | 162 | 1.31 | 2.0e-03 |
| pTH8745 |  | attf-1 | 80 | 1.59 | 2.1e-03 |
| YER148W\_798 |  | che-1 (0.52) K11D2.4 tbp-1 | 188 | 1.27 | 2.1e-03 |
| Spdef |  | lin-1 | 160 | 1.32 | 2.1e-03 |
| pTH6508 |  | nhr-36 | 189 | 1.26 | 2.4e-03 |
| pTH4325 |  | ceh-18 | 115 | 1.43 | 2.5e-03 |
| pTH8982 |  | ceh-48 | 180 | 1.28 | 2.6e-03 |
| MA0117.1 |  | pax-2 F45H11.6 | 145 | 1.34 | 2.6e-03 |
| pTH5887 |  | lin-39 | 140 | 1.35 | 3.1e-03 |
| Hnf4a\_2640 |  | nhr-62 | 138 | 1.35 | 3.2e-03 |
| pTH9974 |  | hlh-32 hlh-16 ngn-1 | 171 | 1.29 | 3.3e-03 |
| K562\_ETS1\_HudsonAlpha |  | lin-1 nhr-28 tbx-39 odr-7 | 276 | 1.14 | 4.2e-03 |
| Pou2f1\_3081 |  | ceh-18 | 142 | 1.33 | 4.5e-03 |
| pTH10034 |  | nhr-66 | 157 | 1.30 | 4.6e-03 |
| MA0541.1 |  | efl-1 F49E12.6 | 166 | 1.29 | 4.7e-03 |
| Plagl1\_0972 |  | Y53H1A.2 | 108 | 1.42 | 4.9e-03 |
| I$DFD\_01 |  | lin-39 | 119 | 1.38 | 5.5e-03 |
| MA0139.1 |  | F58G1.2 | 117 | 1.39 | 5.6e-03 |
| KLF8\_f1 |  | klf-1 | 139 | 1.33 | 5.9e-03 |
| MA0131.1 |  | F39B2.1 | 135 | 1.34 | 6.6e-03 |
| pTH6497 |  | lin-31 (0.69) | 265 | 1.15 | 8.0e-03 |
| pTH6636 |  | egl-5 (0.62) | 134 | 1.33 | 9.0e-03 |
| pTH5891 |  | nhr-49 | 88 | 1.47 | 9.1e-03 |
| pTH6486 |  | nhr-145 | 198 | 1.22 | 9.7e-03 |
| pTH6106 |  | nhr-182 | 133 | 1.33 | 1.0e-02 |
| PAX8\_f1 |  | pax-2 | 140 | 1.31 | 1.0e-02 |
| pTH5924 |  | nhr-255 | 165 | 1.26 | 1.1e-02 |
| pTH9173 |  | efl-2 | 116 | 1.37 | 1.1e-02 |
| pTH9884 |  | tbx-39 | 155 | 1.28 | 1.2e-02 |
| pTH5916 |  | efl-2 | 128 | 1.33 | 1.3e-02 |
| pTH5065 |  | hlh-30 | 143 | 1.30 | 1.3e-02 |
| pTH8216 |  | Y116A8C.22 | 153 | 1.28 | 1.3e-02 |
| ZBTB49\_1 |  | C46E10.9 | 165 | 1.26 | 1.3e-02 |
| V$CEBP\_01 |  | C48E7.11 | 193 | 1.21 | 1.5e-02 |
| pTH9085 |  | nhr-42 | 37 | 1.88 | 1.6e-02 |
| pTH8863 |  | hmg-12 | 246 | 1.15 | 1.6e-02 |
| pTH3046 |  | Y116A8C.22 | 170 | 1.24 | 1.7e-02 |
| pTH6268 |  | ceh-2 | 108 | 1.37 | 1.7e-02 |
| MA0027.1 |  | ceh-16 | 132 | 1.30 | 2.0e-02 |
| Hmbox1\_2674 |  | hmbx-1 | 125 | 1.31 | 2.3e-02 |
| Tcf1\_2666 |  | hmbx-1 | 253 | 1.14 | 2.5e-02 |
| pTH8556 |  | pax-2 | 21 | 2.32 | 2.6e-02 |
| PTF1A\_f1 |  | lin-32 | 182 | 1.21 | 2.8e-02 |
| pTH10768 |  | med-2 | 105 | 1.35 | 2.8e-02 |
| Hey\_SANGER\_5\_FBgn0027788 |  | lin-22 | 137 | 1.28 | 3.0e-02 |
| V$PAX2\_02 |  | pax-1 | 166 | 1.23 | 3.3e-02 |
| pTH9108 |  | daf-12 (0.53) | 206 | 1.18 | 3.3e-02 |
| Nkx3-1\_2923 |  | ceh-24 | 145 | 1.26 | 3.5e-02 |
| V$NCX\_01 |  | ceh-19 | 139 | 1.26 | 4.4e-02 |
| Tcfap2a\_2337 |  | aptf-1 | 69 | 1.46 | 4.6e-02 |

### Correlated (and anti-correlated) transcription factors

|  |  |
| --- | --- |
| **Transcription factor** | **Correlation** |
| nhr-43 | 0.89 |
| moe-3 | 0.87 |
| hlh-33 | 0.82 |
| B0310.2 | 0.82 |
| nhr-114 | 0.80 |
| nhr-260 | 0.80 |
| nhr-270 | 0.79 |
| elt-3 | 0.75 |
| nhr-147 | 0.75 |
| ztf-30 | 0.72 |
| dsc-1 | 0.72 |
| mec-3 | 0.71 |
| nhr-57 | 0.71 |
| nhr-109 | 0.69 |
| lin-31 | 0.69 |
| R07E5.5 | 0.69 |
| hlh-8 | 0.69 |
| npax-2 | 0.68 |
| mls-1 | 0.67 |
| nhr-218 | 0.67 |
| nhr-69 | 0.67 |
| nhr-120 | 0.66 |
| cog-1 | 0.64 |
| hlh-1 | 0.63 |
| Y41D4B.26 | 0.62 |
| wrm-1 | -0.46 |
| hmg-3 | -0.47 |
| repo-1 | -0.47 |
| zim-2 | -0.48 |
| ccch-3 | -0.49 |
| F21G4.5 | -0.49 |
| Y60A9.3 | -0.49 |
| nhr-246 | -0.49 |
| K09A11.1 | -0.49 |
| tbx-36 | -0.49 |
| nhr-220 | -0.49 |
| Y57A10A.31 | -0.50 |
| C28G1.4 | -0.51 |
| zim-3 | -0.53 |
| pqn-75 | -0.53 |
| Y53F4B.5 | -0.53 |
| ztf-15 | -0.55 |
| cep-1 | -0.55 |
| D2030.7 | -0.56 |
| ets-7 | -0.58 |
| Y54G2A.20 | -0.61 |
| pzf-1 | -0.65 |
| ztf-4 | -0.67 |
| snu-23 | -0.67 |
| nhr-276 | -0.68 |

### ChIP peaks enriched

|  |  |  |  |  |
| --- | --- | --- | --- | --- |
| **Gene** | **Experiment** | **Number of upstream peaks** | **Enrichment** | **FDR corrected p** |
| nhr-28 | NHR-28\_Larvae-L4-stage | 89 | 2.37 | 1.0e-12 |
| elt-3 | ELT-3\_Embryos | 58 | 3.09 | 2.9e-12 |
| nhr-23 | NHR-23\_Larvae-L3-stage | 80 | 2.48 | 3.3e-12 |
| nhr-21 | NHR-21\_Larvae-L2-stage | 29 | 3.85 | 7.4e-08 |
| nhr-25 | NHR-25\_Larvae-L2-stage | 62 | 2.25 | 1.3e-07 |
| elt-1 | ELT-1\_Larvae-L2-stage | 23 | 4.33 | 4.3e-07 |
| ama-1 | AMA-1\_Larvae-L3-stage | 37 | 2.48 | 2.7e-05 |
| sax-3 | SAX-3\_Larvae-L4-stage | 68 | 1.72 | 2.8e-04 |
| sem-4 | SEM-4\_Larvae-L2-stage | 62 | 1.74 | 5.6e-04 |
| F23F12.9 | F23F12.9\_Embryos | 17 | 3.08 | 2.2e-03 |
| elt-1 | ELT-1\_Larvae-L3-stage | 22 | 2.21 | 1.7e-02 |
| nhr-76 | NHR-76\_Larvae-L3-stage | 26 | 1.95 | 3.2e-02 |
